# Chemiosmotic ATP synthesis by minimal protocells

**DOI:** 10.1016/j.xcrp.2025.102461

**Published:** 2025-03-19

**Authors:** Fanchen Yu, Jinbo Fei, Yi Jia, Tonghui Wang, William F. Martin, Junbai Li

**Affiliations:** 1 Beijing National Laboratory for Molecular Sciences (BNLMS), CAS Key Lab of Colloid, Interface and Chemical Thermodynamics, Institute of Chemistry, Chinese Academy of Science, Beijing 100190, China; 2 Institute of Molecular Evolution, University of Düsseldorf, 40225 Düsseldorf, Germany; 3 University of Chinese Academy of Sciences, Beijing 100049, China

**Keywords:** origin of life, supramolecular chemistry, nanoarchitectonics, protocell, membrane bioenergetics, hydrothermal vent chemistry, ATP synthesis, gradients

## Abstract

Energy conservation is crucial to life’s origin and evolution. The common ancestor of all cells used ATP synthase to convert proton gradients into ATP. However, pumps generating proton gradients and lipids maintaining proton gradients are not universally conserved across all lineages. A solution to this paradox is that ancestral ATP synthase could harness naturally formed geochemical ion gradients with simpler environmentally provided precursors preceding both proton pumps and biogenic membranes. This runs counter to traditional views that phospholipid bilayers are required to maintain proton gradients. Here, we show that fatty acid membranes can maintain sufficient proton gradients to synthesize ATP by ATP synthase under the steep pH and temperature gradients observed in hydrothermal vent systems. These findings shed substantial light on early membrane bioenergetics, uncovering a functional intermediate in the evolution of chemiosmotic ATP synthesis during protocellular stages postdating the ATP synthase’s origin but preceding the advent of enzymatically synthesized cell membranes.

## Highlights

• Fatty acid membranes can maintain sufficient proton gradients
• ATP synthase-embedded minimal protocells produce ATP
• The protocell membranes have an influence on ATP synthesis

## Graphical abstract

**Figure:**
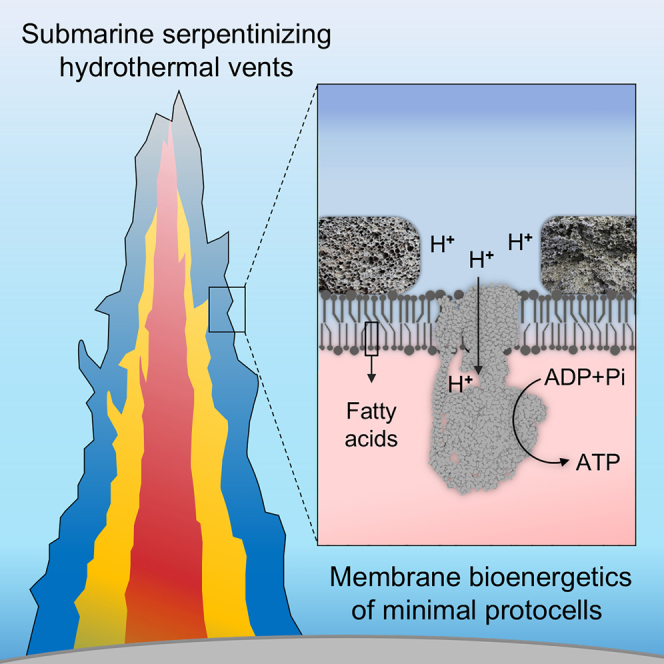

## Teaser

Yu et al. report that fatty acid membranes can maintain sufficient proton gradients, which drive ATP synthase to produce ATP. The chemiosmotic ATP synthesis by these minimal protocells sheds substantial light on early evolutionary intermediates of membrane bioenergetics.

## Introduction

**Scheme 1:**
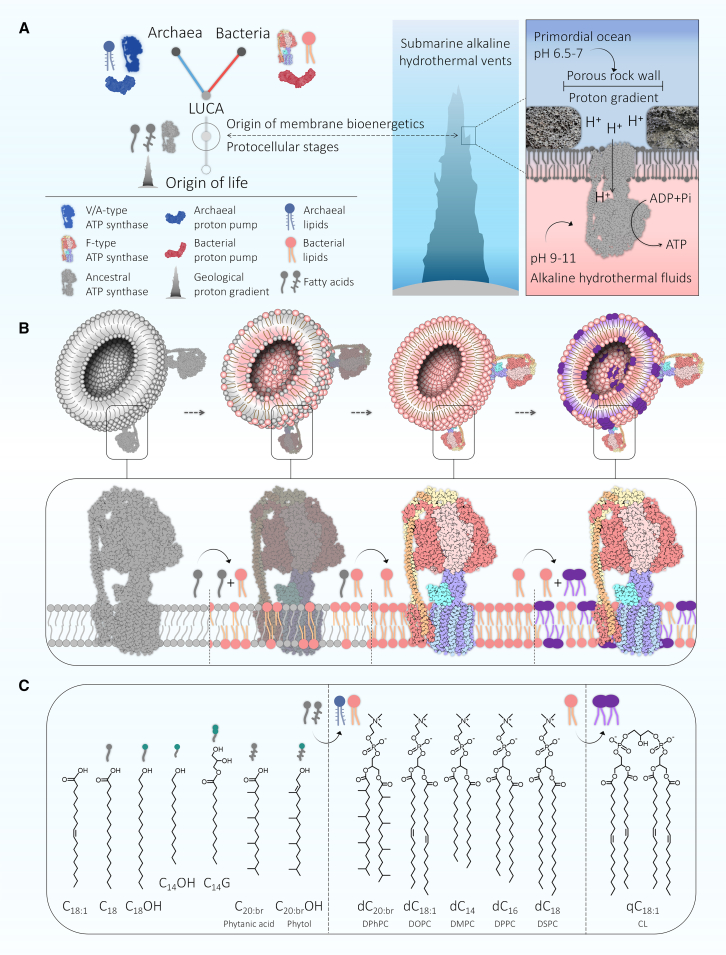
The possible evolution of membrane bioenergetics and its conceptual model protocells (A) The evolutionary relationship of bacteria to archaea suggests that prior to the last universal common ancestor (LUCA), early life went through a protocellular stage with ATP synthase and fatty acid membranes but without proton pumps. In this stage, the ATP synthase could have been driven by geochemical proton gradients across the interface between the primordial oceans (pH = 6.5–7) and the alkaline hydrothermal fluid (pH = 9–11) of serpentinizing hydrothermal vents. (B) At the onset of membrane bioenergetics, a simple fatty acid membrane can maintain proton gradients to drive the ATP synthesis via the ATP synthase. Subsequent adaptation to the free-living lifestyle fosters the transition from single-chain fatty acids to double-chain and quadruple-chain phospholipids. (C) The structural formula of building blocks. C_18:1_, oleic acid; C_18_, stearic acid; C_18_OH, stearyl alcohol; C_14_OH, myristyl alcohol; C_14_G, 1-monomyristoyl glycerol; C_20:br_, phytanic acid; C_20:br_OH, phytol; dC_20:br_, DPhPC; dC_18:1_, DOPC; dC_14_, DMPC; dC_16_, DPPC; dC_18_, DSPC; qC_18:1_, CL, cardiolipin.

Experimental evidence for the processes of energy conservation in the first cells on Earth is scarce, but top-down comparative studies^1^^,^^2^^,^^3^^,^^4^ combined with the bottom-up construction of bio-like nanoarchitectures^5^^,^^6^^,^^7^^,^^8^^,^^9^^,^^10^^,^^11^ render the problem tractable. Though ATP is the universal energy currency for virtually all biochemical or cellular activities that require energy,^12^^,^^13^ its synthesis is afforded by a single molecular species, the ATP synthase, which converts ADP and phosphate into ATP using proton-motive force across phospholipid membranes.^7^^,^^14^^,^^15^^,^^16^^,^^17^^,^^18^^,^^19^ The ATP synthase is as universally conserved as ribosomes and genetic code, while proton pumps that generate the ion gradients it requires are not.^20^ This suggests that the ATP synthase appeared before the last universal common ancestor (LUCA) of all cells diverged into bacteria and archaea^4^^,^^20^^,^^21^^,^^22^ (Scheme 1A).

Bacteria and archaea are located at roots of the Tree of Life,^23^^,^^24^ and their ATP synthase is conserved. But these two prokaryotic domains have distinct membrane molecules structures^20^^,^^25^ and lipid synthesis pathways.^26^^,^^27^ Bacterial phospholipid tails are straight-chain fatty acids (mainly 18 carbons), while archaeal tail chains are branched-chain isoprenoids (mainly C_20_ phytanyl chains).^28^^,^^29^ Their unrelated biosynthetic pathways^20^^,^^25^ suggest that protocells before the LUCA had simpler primitive lipids,^30^ like single-chain fatty acids^31^^,^^32^^,^^33^^,^^34^^,^^35^ or isoprenoid acids,^30^^,^^31^^,^^36^ rather than double-chain phospholipid glycerol conjugates (Scheme 1A). Fatty-acid-based protocells are, however, thought to be unable to support the chemiosmotic ATP synthesis^37^^,^^38^ because primitive membranes assembled from short-chain or unsaturated fatty acids have high membrane fluidity and are “leaky”; that is, they are permeable to small molecules^33^ and/or protons^39^ and, hence, unable to maintain stable ion gradients.

Serpentinizing hydrothermal systems provide an environment highly conducive to chemiosmotic energy conservation.^20^^,^^40^ Since there was first water on Earth, serpentinizing hydrothermal vents have continuously forced warm (40°C–100°C)^41^^,^^42^^,^^43^^,^^44^ alkaline water (pH = 9–11)^45^ to interface with ocean water (pH = 6.5–7),^46^ generating stable, natural, geochemical proton and temperature gradients^40^^,^^47^^,^^48^^,^^49^ (Scheme 1A). These proton gradients could, in principle, serve as the evolutionary precursor of biological proton pumps. However, this requires that protocells with abiotically primitive lipid membranes could harness such geochemically formed pH gradients.^4^ Heat flux generated by temperature gradients of hydrothermal vents contributes to thermophoretic enrichment and the assembly of amino acids, nucleotides, and, importantly, lipids.^50^ Yet, the ATP synthase requires a proton-tight membrane of hydrophobic molecules with the thickness of an ATP synthase membrane subunit to function.^7^^,^^51^^,^^52^ Fatty acids up to 18 carbons are synthesized from H_2_ and CO_2_ with simple metal catalysts under conditions of hydrothermal vents, providing a source of primordial lipid monomers.^53^^,^^54^^,^^55^^,^^56^^,^^57^ However, the crucial question of whether membranes consisting of such simple, abiotically formed, straight-chain lipids can support ATP synthase function has not been experimentally answered to date.

Here, we show that membranes consisting solely of solitary long-chain saturated fatty acids maintain proton gradients that power an ATP synthase to produce ATP in a minimal protocell (Scheme 1B). Structures of membrane molecules and temperature in the assembled system can modulate membrane assembly, its ability to maintain proton gradients, fluidity and ATP synthesis. The results uncover an evolutionary intermediate in primordial bioenergetics linking ATP synthase function in abiotic fatty acid membranes using geochemically formed gradients to ATP synthesis in biochemically synthesized phospholipid bilayers.

## Results and discussion

### Long-chain saturated fatty acid membranes outperform some phospholipid membranes in pH gradient stability

We began by investigating proton gradients. Vesicles containing a pH fluorescent probe (8-hydroxypyrene-1, 3, 6-trisulfonic acid trisodium salt [HPTS])^19^ were obtained (Figure S1). According to the hydrothermal vent theory,^1^^,^^2^^,^^4^^,^^40^ protocells harnessed natural geological proton gradients for ATP synthesis.^20^ Therefore, we formed analogous proton gradients using the acid bath method, changing pH outside the vesicles from about pH 9.5 to 6.5 (Figure S2). Because the chain lengths of phospholipids in modern cell membranes are typically 18 carbons,^58^ we mainly chose building blocks with similar chain lengths (Scheme 1C). Consistent with previous studies, long-chain unsaturated oleic acid (C_18:1_) vesicles alone cannot maintain a proton gradient^39^; their spectra coincide within 1 min after an acid bath and after the addition of detergent TX-100, breaking the vesicles (Figure S3A). A confocal laser scanning microscope (Figure S3B) and dynamic light scattering (Figure S4) show that the acid bath did not directly break C_18:1_ vesicles (Figure S5).

**Figure 1:**
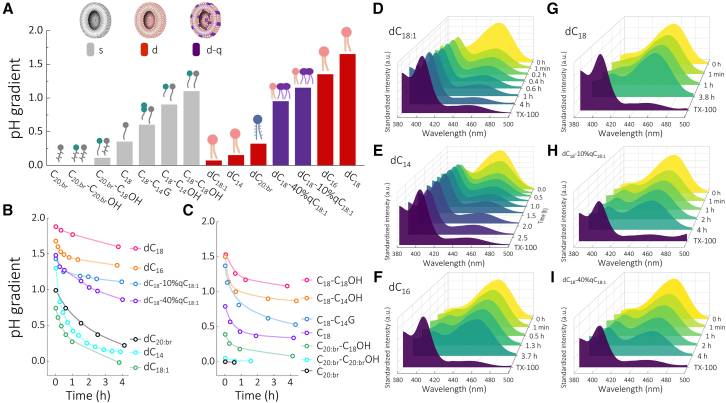
Proton permeability of membranes assembled with fatty acids or phospholipids (A) pH gradients of vesicles after acid bath for 3 h at room temperature (RT; ∼20°C). The molar ratio of fatty acid/alcohol is 2:1. s, single chain (gray); d, double chain (red); d-q, double chain and quadruple chain (purple). (B and C) pH gradients of vesicles composed of phospholipids (B) or fatty acids and their derivatives (C) over time at RT. Their first point of curves starts at 1 min after an acid bath, considering that the pH jump occurs within 1 min, due to residual HPTS outside vesicles and electrically uncompensated proton influx.^61^^,^^62^ Next, there are two phases of fast and slow pH decay due to the transient-pore mechanism, solubility-diffusion mechanism, and counterion flux limitation.^63^ (D–I) Excitation spectra of HPTS inside vesicles composed of phospholipids after acid bath over time at RT. After adding TX-100 to break vesicles, the pH outside the vesicles was obtained. (D) dC_18:1_, (E) dC_14_, (F) dC_16_, (G) dC_18_, (H) dC_18_-10%qC_18:1_, and (I) dC_18_-40%qC_18:1_.

Next, we tested long-chain saturated fatty acid (C_18_) vesicles as a reference for subsequent comparisons. Numerous C_18_ vesicles were still observed after spending 2 days in an acid bath (Figure S6). We found that simple C_18_ vesicles maintain a pH gradient over 0.35 pH units after a 3 h acid bath (Figures 1A and S7), a pH gradient that is within the range of 0.3–0.5 pH units measured for growing *E. coli*.^59^^,^^60^ In contrast, the pH gradient of unsaturated double-chain phospholipid vesicles with the same chain length (dC_18:1_) approaches 0 after 3 h, but its pH gradient did not dissipate within 1 min like C_18:1_ vesicles (Figures 1B and 1D). Moreover, the pH gradient of saturated dC_18_ is still over 1.6 pH units, approximately 5 times that of C_18_ vesicles (Figures 1A and 1G). These findings show that doubling the chain number or increasing the degree of saturation improves the ability of protocell fatty acid membranes to maintain proton gradients.

The presence of two hydrophobic chains linked to glycerol per lipid monomer is a strictly conserved feature of both bacterial and archaeal membranes^25^^,^^26^^,^^27^^,^^28^^,^^29^^,^^31^ and, hence, an important evolutionary advance from protocell-type to enzymatically synthesized membranes. To investigate the effect of further doubling the chain number, we tested a representative cardiolipin (qC_18:1_). qC_18:1_ has four unsaturated tail chains, located in the mitochondrial inner membrane and in some bacteria, where its content reaches up to 10%–20%.^64^ The results show that increasing the molar ratio of qC_18:1_ in dC_18_ vesicles increases proton permeability (Figure 1A), while the pH gradients are still higher compared to those in C_18_ and dC_18:1_ vesicles. Cardiolipin is not known to be conducive to maintaining proton gradients but exerts regulatory roles instead.^64^

To explore the interval of proton permeability required to maintain a proton gradient capable of energy conversion in protocells, we tested phospholipids with shorter chain lengths. When the phospholipid chain length decreases to 14 carbons (dC_14_), the ability to maintain a proton gradient is lower than in C_18_ vesicles (Figure 1A). Reconstituted ATP synthase in dC_14_ glycerol ester phospholipid vesicles has previous been studied.^7^^,^^14^^,^^15^^,^^16^^,^^17^^,^^18^^,^^19^

Mixing fatty alcohols into fatty acid vesicles can effectively enhance membrane stability^31^^,^^65^ and enhance proton gradient ‌maintenance (Figure 1). Increasing the chain length of fatty alcohols can also decrease membrane permeability. Membrane permeability of fatty acid glycerides is greater than that of fatty alcohols, likely due to the larger glycerol head group, which decreases membrane tightness.

Our experiments so far have employed bacterial-type aliphatic chains as hydrophobic components. To probe the ability of archaeal-type hydrophobic tails, we generated vesicles composed of branched-chain fatty acids. They failed, however, to maintain the proton gradients, although the vesicles themselves were still observed after acid bath treatment (Figures 1 and S8). Their pH gradients dissipated within 1 min. It is more likely that archaeal-type fatty acids are produced by biological synthesis at archaeal stages rather than by abiotic processes.^25^^,^^26^^,^^27^^,^^28^^,^^29^^,^^31^ Our results indicate that protocell membranes composed of abiotically synthesized straight-chain fatty acids can maintain proton gradients. The fatty acids, synthesized from H_2_ and CO_2_ in serpentinizing systems,^53^^,^^54^^,^^55^^,^^56^ could have served to maintain geochemical ion gradients in the common ancestor of archaea and bacteria.^4^^,^^20^

### Protocell membrane bioenergetics require temperature gradients

**Figure 2:**
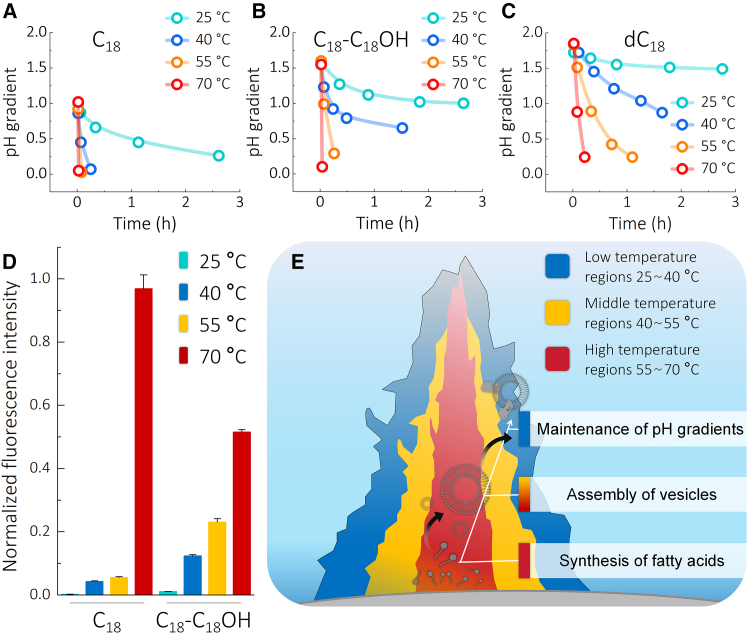
The effect of temperature on the proton gradients and formation of protocells (A–C) The pH gradients of (A) C_18_, (B) C_18_-C_18_OH, and (C) dC_18_ vesicles over time at different temperatures: 25°C (cyan), 40°C (blue), 55°C (yellow), and 70°C (red). (D) Fluorescence intensity at 460 nm of C_18_ and C_18_-C_18_OH vesicles prepared at different temperatures: 25°C (cyan), 40°C (blue), 55°C (yellow), and 70°C (red). Data are represented as mean ± SEM. (E) Scheme of the roles of temperature gradients in alkaline hydrothermal vents for protocells. High-temperature regions contribute to the synthesis of fatty acids and the formation of vesicles, while relatively low-temperature regions allow maintaining proton gradients.

Temperature affects the formation of long-chain saturated fatty acid vesicles^66^ and their membrane permeability. C_18_ vesicles at 40°C, C_18_-C_18_OH vesicles at 55°C, and dC_18_ vesicles at 70°C lose proton gradients within 15 min (Figures 2A–2C). However, vesicle formation is improved at 70°C, and the numbers of obtained vesicles containing fluorescence pH probes decreases sharply with decreasing temperature (Figures 2D and S9). The precipitation of C_18_ or C_18_-C_18_OH was not significant at 1 mM, and numerous vesicles were still observed after cooling down from 70°C to room temperature (RT) (Figure S10).

While the formation of long-chain saturated fatty acid vesicles requires higher temperatures (70°C), maintaining proton gradients requires lower temperatures (40°C). The requirement of different temperature ranges for thermophoretic concentration mechanisms,^50^ vesicle formation, and ion gradient maintenance implicate an environment with temperature gradients as the site of bioenergetic origin.^67^ The naturally existing temperature gradients (40°C–75°C)^41^ in vents of serpentinizing hydrothermal systems^68^ satisfy the temperature, ion gradient, and lipid monomer synthesis conditions required for ATP synthase function (Figure 2E).

The temperature of primordial ocean is still discussed. Some argue that primordial ocean reached up to 70°C 3.5 billion years ago,^42^ while recent findings suggest that primordial oceans had more mild temperatures.^44^ Our results show that both fatty acid and phospholipid vesicles fail to maintain proton gradients at high temperatures, which would preclude their function in chemiosmotic ATP synthesis. This suggests that prior to the origin of enzymatic lipid synthesis, the transition from soluble energy-conserving reactions to energy conservation with an ATP synthase^4^^,^^20^ required a mild temperature range.

### Protocells reveal a trade-off between membrane fluidity and proton permeability for improving ATP synthesis

**Figure 3:**
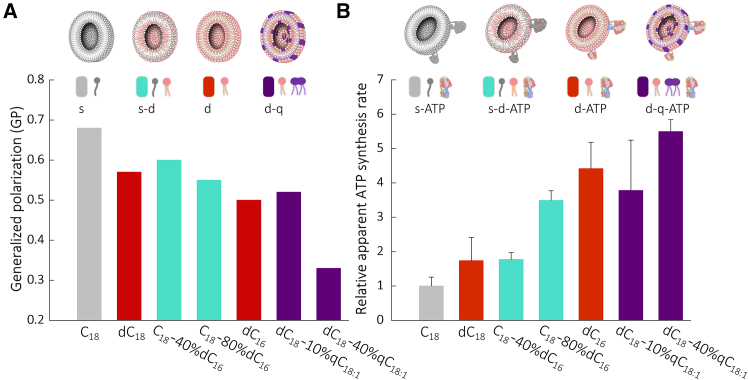
ATP bioenergy synthesis of model protocells reconstituted with ATP synthase (A) Fluidity of membranes assembled with fatty acids or phospholipids. The GP value of vesicles at room temperature (RT; ∼20°C). s, single chain (gray); s-d, single chain and double chain (cyan); d, double chain (red); d-q, double chain and quadruple chain (purple). (B) Relative apparent ATP synthesis rate of vesicles reconstituted with ATP synthase after base bath at RT. The rate is calculated based on the slope of the initial 100 s of ATP production over time, using the rate of C_18_ as the reference. s-ATP, single-chain fatty acids reconstituted with ATP synthase (gray); s-d-ATP, single-chain fatty acids and double-chain phospholipids reconstituted with ATP synthase (cyan); d-ATP, double-chain phospholipids reconstituted with ATP synthase (red); d-q-ATP, double-chain and quadruple-chain phospholipids reconstituted with ATP synthase (purple). Data are represented as mean ± SEM.

Membrane fluidity is another key factor impacting membrane protein function.^69^^,^^70^^,^^71^ We tested the membrane fluidity of vesicles using a fluorescence probe (Laurdan).^72^^,^^73^ A lower generalized polarization (GP) value indicates higher membrane fluidity. Usually, the GP value of normal cell membranes is about 0.2–0.8.^72^ Membrane fluidity increases with the decreasing chain length, and the introduction of qC_18:1_ has a similar affect (Figures 3A and S11). Usually, lower membrane fluidity means lower permeability (Figures 1 and 3A), but when comparing C_18_ with dC_18_, doubling the chain number can simultaneously improve membrane fluidity and the ability to maintain proton gradients.

Generally, when the GP value falls below 0.3, the membranes are in a liquid state.^74^ The fatty acid vesicles (C_18:1_, C_20:br_, and C_20:br_-C_20:br_OH at RT and C_18_ and C_18_-C_18_OH at 70°C) at a liquid state cannot maintain proton gradients, while the phospholipid vesicles (dC_18:1_, dC_20:br_, and dC_14_ at RT and dC_18_ at 55°C) at a liquid state can do those (Figures 1, 2, S11, and S12).

We examined protocell membranes for their ability to support and modulate ATP synthesis using reconstituted ATP synthase (Figures 3B and S13). The results show that ATP synthase is functional in fatty acid membranes. The ATP synthesis rate increases with decreasing chain length and increasing chain number. Increasing the molar ratio of phospholipid (dC_16_) in fatty acid (C_18_) vesicles or cardiolipin (qC_18:1_) in phospholipid (dC_18_) vesicles increases the ATP synthesis rate. The ATP synthesis rate for dC_16_ vesicles is 4.4 times compared to that for C_18_ vesicles. Meanwhile, the pH gradient at 3 h for the former is 3.8 times higher than that for the latter. These findings indicate the impact of protocell membrane components on protocell energy metabolism.

The ATP synthesis rate and membrane fluidity show a positive correlation (Figure 3), possibly because lower membrane fluidity imparts greater obstruction to rotary catalysis of ATP synthase, leading to a decrease in the ATP synthesis rate. The increase of chain length in fatty acids maintains higher proton gradients but decreases membrane fluidity in corresponding vesicles (Figures 1A and 3A). This suggests a trade-off in protocell membrane fluidity and proton permeability, limiting the lipid chain length range in protocells that support ATP synthase function, even in the presence of a large proton gradient.

In summary, we have shown that protocells enclosed by simple fatty acid membranes can maintain ion gradients and support ATP synthesis via a rotor-stator ATP synthase. The findings show that interactions between complex proteins and abiotically synthesized fatty acids can support membrane bioenergetics via harnessing natural geological proton gradients generated by serpentinization at hydrothermal vents^1^^,^^2^^,^^20^^,^^40^ (Scheme 1A). Observed differences between permissive temperatures for the formation of vesicles and maintenance of proton gradients may indicate that membrane bioenergetics originated in environments with natural gradients^67^ rather than in isotropic settings (Figure 2E).

Fatty acid composition in protocell membranes specifies membrane fluidity and proton permeability, properties that influence ATP synthase function, also in modern cells.^51^^,^^52^^,^^75^ The evolutionary transition from abiotically synthesized fatty acid membranes to enzymatically synthesized phospholipids with two chains per monomer improved membrane fluidity and proton gradient stability to a level that has not been improved in 4 billion years, barring the appearance of tetraether lipids in some thermophilic archaea.^29^

The ATP synthase, one of the most sophisticated proteins known,^75^ could function in protocellular lipids before free-living cells arose. This may explain how it is possible that bacteria and archaea share the ATP synthase but independently evolved their biosynthetic pathways for membrane lipids^25^: the primordial ATP synthase might not have required enzymatically synthesized lipids to function. That such a complex protein is so ancient might seem to present a paradox.^76^^,^^77^ A possible scenario is serpentinizing hydrothermal vents where complex ATP synthase and simple lipids were present before the emergence of free-living cells.^1^^,^^2^^,^^3^^,^^4^^,^^20^^,^^21^^,^^22^ Primitive lipids (such as fatty acids) could be synthesized from H_2_ and CO_2_ by geological catalysts in serpentinizing hydrothermal vents.^53^^,^^55^ These primitive lipids self-assembled into membranes for embedding ATP synthase translated by ribosomes.^78^ Then, ATP was produced by ATP synthase driven by geological proton gradients of serpentinizing hydrothermal vents as bioenergy currency to fuel the enzymatic synthesis of complex lipids^26^^,^^27^ and other biochemical activities. Although the catalytic function of individual proteins,^79^^,^^80^ and even entire enzymatic pathways,^81^ can be replaced by inorganic catalysts of serpentinizing hydrothermal systems, the ATP synthase function in protocellular lipids represents a special case: its rotor-stator catalytic mechanism has no inorganic or environmental precursor, while the ion gradient that powers it does. Among the many environments that have been suggested for the origin of biological systems,^47^^,^^48^^,^^49^^,^^50^^,^^67^^,^^68^ serpentinizing hydrothermal systems are unique in that they generate natural proton gradients^1^^,^^2^^,^^3^^,^^4^^,^^20^^,^^21^^,^^22^ that can power an ATP synthase in primitive fatty acid membranes, connecting Earth chemistry and life chemistry in energy conservation.^20^^,^^40^

## Methods

Details regarding the methods can be found in the supplemental information.

## Resource availability

### Lead contact

Requests for further information and resources should be directed to and will be fulfilled by the lead contact, Junbai Li (jbli@iccas.ac.cn).

### Materials availability

This study did not generate new unique reagents.

### Data and code availability

• All data reported in this paper will be shared by the lead contact upon request.
• This paper does not report original code.
• Any additional information required to reanalyze the data reported in this paper is available from the lead contact upon request.

## Acknowledgments

This work was funded by the 10.13039/501100001809National Natural Science Foundation of China (no. 22193031). W.F.M. acknowledges funding from the 10.13039/501100000781European Research Council (no. 10118894).

## Author contributions

Conceptualization, J.L. and F.Y.; supervision, J.L.; methodology, J.L., F.Y., W.F.M., and J.F.; experiments, F.Y. and T.W.; data curation, analysis, and visualization, J.L., F.Y., W.F.M., and J.F.; writing – original draft, F.Y.; writing – review & editing, J.L., W.F.M., Y.J., J.F., and F.Y.

## Declaration of interests

The authors declare no competing interests.

## Supplemental information

Document S1. Figures S1–S13 and supplemental methods

Document S2. Article plus supplemental information
