# Supplementary material for: Chemiosmotic ATP synthesis by minimal protocells
Source: Cell Rep Phys Sci. 2025 Mar 19;6(3):102461. doi: 10.1016/j.xcrp.2025.102461 (PMC11922820; doi:10.1016/j.xcrp.2025.102461)
Supplement: Document S1. Figures S1–S13 and supplemental methods [file mmc1.pdf]

Cell Reports Physical Science, Volume *6*

Supplemental information

Chemiosmotic ATP synthesis by minimal protocells

Fanchen Yu, Jinbo Fei, Yi Jia, Tonghui Wang, William F. Martin, and Junbai Li

Table of Contents

Supplemental Methods
- Materials
- Vesicles
- pH gradients
- Turbidity
- Membrane fluidity
- Purification of ATP synthase
- Reconstitution of ATP synthase
- Synthesis of ATP
- Characterization

Supplemental Items
- Figure S1. Encapsulation of pH fluorescent probes in vesicles and formation of pH gradients.
- Figure S2. Determination of solution pH through the pH fluorescent probe.
- Figure S3. The $C_{18:1}$ vesicles cannot maintain proton gradient.
- Figure S4. Dynamic light scattering of various vesicles before and after acid bath.
- Figure S5. Resistance of fatty acid vesicles to pH changes.
- Figure S6. The resistance of C_{18} or C_{18}-$C_{18}OH$ vesicles to acid bath.
- Figure S7. Excitation spectra of HPTS inside vesicles after acid bath over time.
- Figure S8. The resistance of other fatty acid vesicles to acid bath.
- Figure S9. The relationship between the formation of vesicles and temperature.
- Figure S10. The influence of temperature and concentration on vesicles
- Figure S11. The GP value of vesicles assembled with fatty acids/alcohols or phospholipids.
- Figure S12. The influence of temperature on membrane fluidity.
- Figure S13. Verification of structure and function of ATP synthase.

Supplemental References

Supplemental Methods

Materials

1,2-dimyristoyl-sn-glycero-3-phosphocholine (dC_{14}, DMPC) was purchased from Avanti. 1,2-dipalmitoyl-sn-glycero-3-phosphocholine (dC_{16}, DPPC) and 1,2-distearoyl-sn-glycero-3-phosphocholine (dC_{18}, DSPC) were purchased from Sigma-Aldrich. 1,2-dioleoyl-sn-glycero-3-phosphocholine ($dC_{18:1}$, DOPC), 1′,3′-bis[1,2-dioleoyl-sn-glycero-3-phospho]-glycerol sodium salt ($qC_{18:1}$, cardiolipin), oleic acid ($C_{18:1}$), tetradecanol ($C_{14}OH$), monomyristin ($C_{14}G$) and phytol ($C_{20:br}OH$) was purchased from Macklin. Stearic acid (C_{18}) was purchased from Aladdin (Shanghai) Reagent Co., LTD. Phytanic acid ($C_{20:br}$) was purchased from Kaiwei Chemical. HPTS was purchased from Acros Organics. Laurdan was purchased from Macklin. 6-tetramethylrhodamine isothiocyanate (TRITC) was purchased from Avanti. Sephadex G-50 was purchased from JandK Scientific. Di(adenosine-5')pentaphosphate trilithium salt (Ap_5A) was purchased from Shanghai yuanye Bio-Technology Co., Ltd. Oligomycin was purchased from Aladdin Reagent (Shanghai) Co., LTD. Ethylenediaminetetracetic acid disodium salt (EDTA-Na_2), sodium dihydrogen phosphate (NaH_2PO_4), magnesium chloride ($MgCl_2$), manganese sulfate ($MnSO_4$) and ammonium hydrogen carbonate (NH_4HCO_3) were purchased from Aladdin Reagent (Shanghai) Co., LTD. Dithiothreitol (DTT) was bought from Merck. Luciferase Assay System was from Promega. Triton X-100, adenosine-5'-triphosphonic acid disodium salt (ATP Na_2), β-D-octyiglucoside and Adenosine-5'-diphosphate disodium salt (ADP Na_2) were purchased from Solarium. SDS-PAGE Gel Preparation Kit was obatined from Beyotime. Coomassie brilliant blue G-250 was from Solarbio Biotechnology Co., LTD. Bio-Beads SM-2 was purchased from BIO-RAD. All chemicals were used directly without further purification. Deionized water (18.2 MΩ·cm) was obtained by ELGA PURELAB (U.K.)

Vesicles

All vesicles were prepared by thin-film hydration method. In detail, each membrane component was dissolved with 2 mL of chloroform in a round-bottom flask. The concentration of fatty-acid tail chain in every vesicle was set at 1 mM. Next, uniform film formed at the bottom of the flask by a rotary evaporator at 55 °C for phospholipids (above the phase transition temperature) or Tm - 10 °C for fatty acids, and was placed in a vacuum over overnight. Then, 2 mL of hydration solution (10 mM K_2HPO_4, 0.1 mM KOH, pH 9.5) was added to keep hydration for 2 h at 70 °C, a temperature of alkaline hydrothermal fluid.[1] The vesicles were repeatedly extruded through the PC membrane with a pore size of 200 nm in the preheated Mini-extruder to obtain uniform unilamellar vesicles for pH gradient and ATP synthesis experiments. The vesicles used for microscopy were not extruded.

pH gradients

In the method of preparing vesicles, 2 mL of hydration solution containing pH probes (10 mM K_2HPO_4, 1 mM KOH, 1 mM HPTS, pH 9.5) was added to keep hydration for 2 h at 70 °C.

To obtain vesicles containing pH probes, 1 mL of vesicle solution with pH probes was took and the probe outside the vesicle was removed by size exclusion chromatography (17mm×13.4mm×305mm glass column filled with Sephadex G-50 medium beads) and elution solution (10 mM K_2HPO_4, 0.1 mM KOH, 2.45 mM KCl, pH 9.5). Then, the fractions were collected by a 96 well plate. To determine the location of vesicles, the absorbance at 450 nm was detected by a microplate reader. Finally, these vesicles containing the pH probes were collected. To observe these vesicles by confocal fluorescent microscopy, 50 µL of vesicles and 0.5 µL of 1 mM TRITC dye were mixed before observation.

To generate pH gradient across the vesicle membrane, 0.2 mL of vesicles and 2 mL of acid bath solution (pH 6.5, 7.5 mM KH_2PO_4, 2.5 mM K_2HPO_4, 6.25 mM KCl) were mixed and stirred for 1 min. Next, the excitation spectrum of the mixture from 380 to 500 nm was measured over time (emission at 513 nm). Finally, 1 µL of Triton X-100 was added to break the vesicles, the excitation spectrum was recorded as the pH value outside the vesicle to calculate the pH gradient, $\Delta pH(t) = pH(t) - pH(Triton)$.

In our case, HPTS was used as the pH probe. As proton concentration increases, the excitation intensity at 406 nm increases while that at 460 nm decreases. The pH can be calculated from the excitation intensity ratio I_{460}/I_{406} according to the standard curve, as the following equation:

$$pH = a + bx + cx^2 + dx^3 + ex^4 + fx^5$$

where x is I_{460}/I_{406}. The constants are a: 6.13, b: 3.20, c: -3.26, d: 2.05, e: -0.64, f: 0.079.

Turbidity

Turbidity titration. Firstly, fatty acids (final concentration 100 mM) were dissolved in 0.1 M NaOH and a few microliters of 2 M HCl were added each time to decrease its pH while shaking it for 5 min. Then, measure its pH value by pH meter and its absorbance at 480 nm by UV-Vis spectrophotometer. Repeat this process until its pH<4.

Acid bath of preformed vesicles. Fatty acids (final concentration 100 mM) were dissolved in 0.15 M KOH to form micelles (pH>12). To form vesicles, 25 μL micelles and 475 μL pH 8.3 buffer (pH 8.3, 100 mM Bicine, 10 mM KH_2PO_4) were mixed in an orbital shaker (25 °C, 90 rpm). Next, mix 0.5 mL vesicles and 1.5 mL pH 6.5 buffer (pH 6.5, 100 mM Bis-Tris, 10 mM KH_2PO_4). Finally, the mixture's absorbance at 480 nm was measured over time.

Acid bath of micelles. Fatty acids (final concentration 100 mM) were dissolved in 0.15 M KOH to form micelles (pH>12). Directly mixing 25 μL micelles, 475 μL pH 8.3 buffer (pH 8.3, 100 mM Bicine, 10 mM KH_2PO_4) and 1.5 mL pH 6.5 buffer (pH 6.5, 100 mM Bis-Tris, 10 mM KH_2PO_4). Finally, the mixture's absorbance at 480 nm was measured over time.

To observe these vesicles by confocal microscopy, 50 μL vesicles and 0.5 μL 1 mM TRITC dye were mixed before observation.

Membrane fluidity

Vesicles were prepared by thin-film hydration method. The membrane components were dissolved by 2 mL chloroform containing 5 μM membrane fluidity probes (Laurdan) in a round-bottom flask. The concentration of fatty-acid tail chain in every vesicle was set at 1 mM. Next, uniform films were obtained at the bottom of the flask by a rotary evaporator at 55 °C for phospholipids and below the melting point by 10 °C for fatty acids. They were placed in a vacuum over overnight. Through the similar procedure, unilamellar vesicles were prepared by repeatedly extruding the vesicles through the PC membrane with a pore size of 200 nm in the preheated Mini-extruder. 2 mL of hydration solution (10 mM K_2HPO_4, 0.1 mM KOH, 2.45 mM KCl, pH 9.5) was added and hydrated for 2 h at 70 °C. Finally, the emission spectrum of the vesicles from 400 to 600 nm was measured (excitation at 370 nm).

According to the probe's emission spectrum, GP (generalized polarization) value is calculated as the following equation:[2]

$$GP = (I_{430} - I_{500}) / (I_{430} + I_{500})$$

The higher GP value means the lower membrane fluidity.

Purification of ATP synthase

ATP synthase was purified from chloroplasts of spinach according to the previous method.[3] Firstly, fresh spinach (500 g) was cleaned thoroughly and stored at 4 °C overnight. After adding buffer I (500 mL, pH 8.0, 2 mM $MgCl_2$, 100 mM Tricine-NaOH, 0.4 M sucrose), the spinach veined was triturated to grainy by homogenizer. This crushed mixture was filtered by cotton gauze, and the filtrate was centrifuged (10600 gmax, 30 min) to collect the precipitation, which was suspended in buffer II (pH 8.0, 0.5 mM $MgCl_2$, 10 mM Tris-HCl). After centrifuging this suspension (16900 gmax,15 min), the precipitation was dispersed in buffer III (pH 8.0, 0.5 mM $MgCl_2$, 0.4 M sucrose, 10 mM Tris-HCl) for the next centrifugation (16900 gmax for 25 min). Then after removal of the supernatant, buffer IV (pH 8.0, 0.2 mM $MgCl_2$, 0.4 M sucrose, 50 mM Tricine-NaOH) was poured into the tube to obtain a chlorophyll solution ~5 mg/mL. And the suspension was mixed with equal volume buffer V (pH 8.0, 50 mM DTT, 60 mM β-D-octylglucoside, 25 mM Na cholate, 2 mM ATP Na_2, 200 mM sucrose, 20 mM Tricine-NaOH, 5 mM $MgCl_2$, 400 mM $(NH_4)_2SO_4$) under stirring for 30min. After centrifugation (208000 gmax, 60 min), the suspension was injected $(NH_4)_2SO_4$ (~45%) to make the ATP synthase precipitate out, and collected by centrifugation (12000 gmax, 10 min). After mixed with buffer VI (pH 7.2, 4 mM dodecylmaltoside, 0.5 mM Na_2EDTA, 2 mM $MgCl_2$, 200 mM sucrose, 30 mM NaH_2PO_4), the crude extract was precipitated and then adding buffer VII (pH 7.2, 4 mM dodecylmaltoside, 0.5 mM Na_2EDTA, 30 mM NaH_2PO_4, 2 mM $MgCl_2$) with equal volume. After sucrose density gradient centrifugation (60%, 52%, 44%, 36%~28%, and 20%), ATP synthase was collected at 44% sucrose layer and stored in liquid nitrogen. Finally, the structure of ATP synthase was verified through SDS-PAGE (sodium dodecyl sulphate-polyacrylamide gel electrophoresis).

Reconstitution of ATP synthase

940 μL of vesicle solution was mixed 80 μL of 10% Triton X-100 and 60 μL of ATP synthase and stirred for 1 h at room temperature. Then, Bio-Beads SM2 was added to remove Triton X-100.[3,4] The ATP synthase reconstituted vesicles were obtained.

Synthesis of ATP

For ATP synthesis, proton gradient was generated by base bath and the ATP concentration was measured by the luciferin-luciferase assay. In detail, 100 μL of base bath solution (10 mM K_2HPO_4, 0.8 mM KOH, 2.45 mM KCl, 0.2 mM ADP, pH 9.5) was mixed with 100 μL of ATP synthase reconstituted vesicles (pH 7.5) and 10 μL of luciferin-luciferase. Next, photon counts of the mixture were detected by using an ultra-weak luminescence analyzer (BPCL-GP15). Finally, ATP concentration was calculated through the standard curve obtained by pure ATP solution. The detection principle of ATP in luciferin-luciferase assay is to consume ATP to emit photons:

$$\text{ATP} + \text{luciferin} \xrightarrow[\text{Mg}^{2+},\ \text{O}_2]{\text{luciferase}} \text{AMP} + \text{oxyluciferin} + \text{H}_2\text{O} + \text{CO}_2 + \mathbf{hv}$$

Characterization

Confocal laser scanning microscopy (CLSM) images were obtained by using an Olympus FV3000. The zeta potential test results were obtained from Zetasizer Nano 7S ZEN3600. ATP production was tested by Luciferin-luciferase assay using the ultra-weak luminescence analyzer (BPCL-GP15). Fluorescence excitation spectra were measured by Edinburgh FLS980 fluorescence spectrometer and Edinburgh FS5 Fluorescence Spectrometer. Absorption spectra were measured by Shimadzu UV-Vis spectrophotometer UV-3600i Plus and Thermo Scientific Muitiskan FC microplate reader.

Supplemental Items

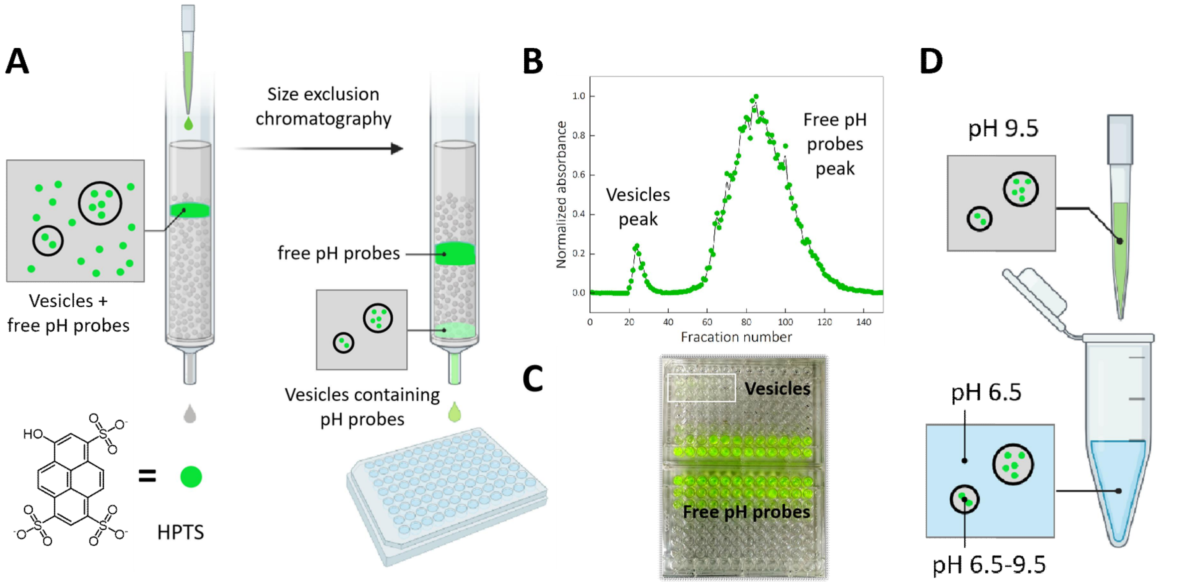

Figure S1. Encapsulation of pH fluorescent probes in vesicles and formation of pH gradients. (A) Schematic diagram of obtaining vesicles containing pH probes. (B) Elution curve of fractions. (C) Collecting fractions based on the vesicle peak of the elution curve. (D) Schematic diagram of forming proton gradients by acid bath.

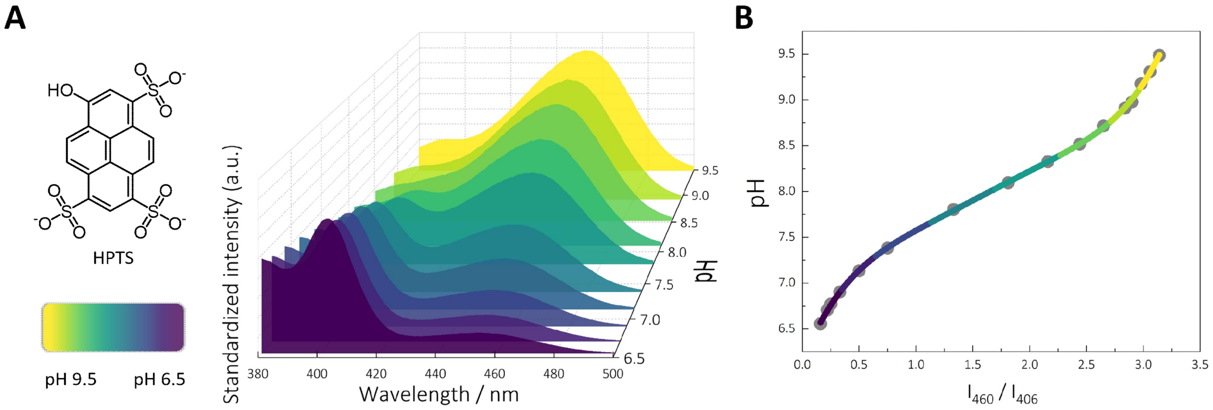

Figure S2. Determination of solution pH through the pH fluorescent probe. (A) Excitation spectra of HPTS over pH. (B) Standard curve of HPTS presenting pH over excitation intensity ratio **I_{460}/I_{406}**.

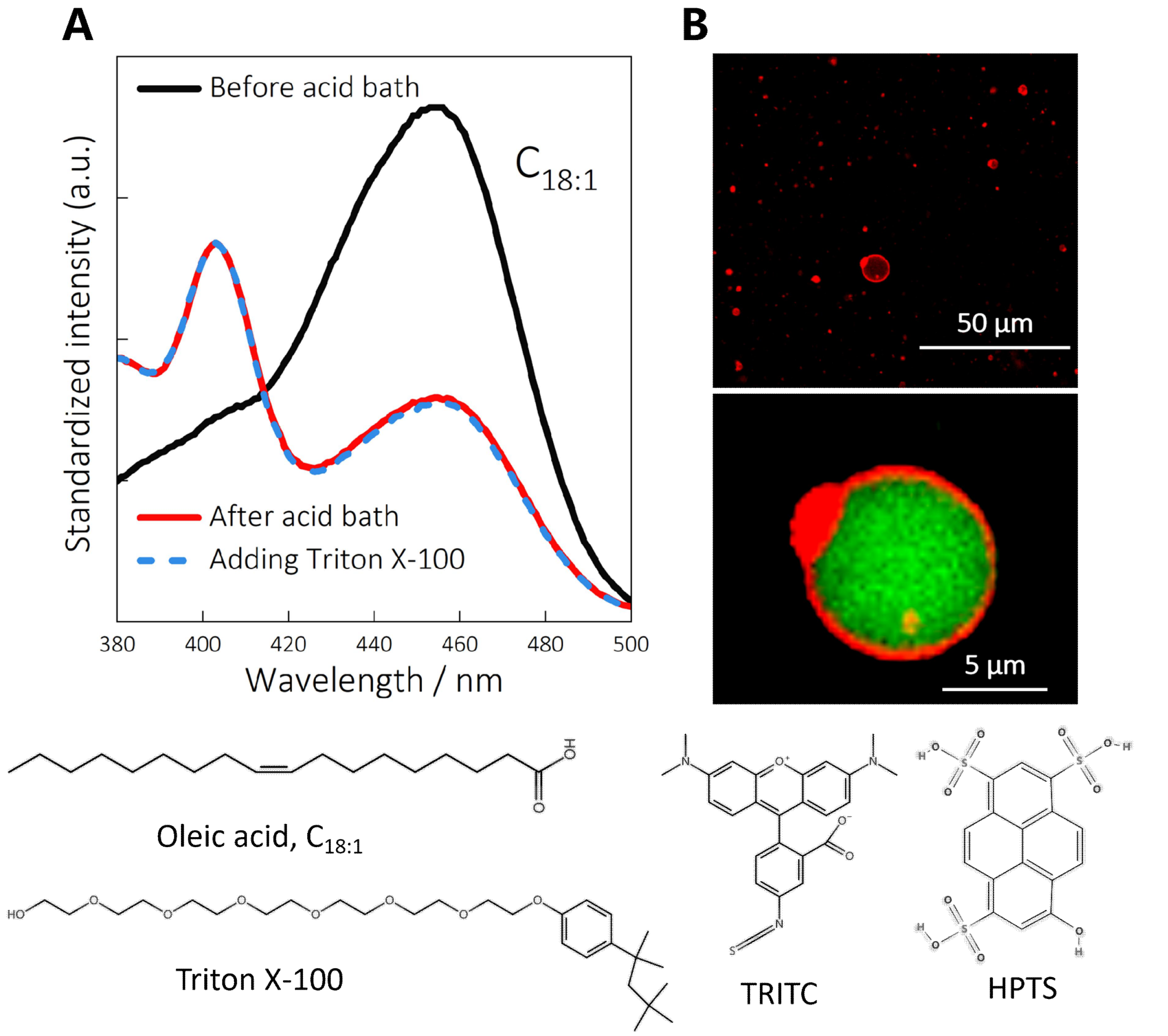

Figure S3. The $C_{18:1}$ vesicles cannot maintain proton gradient. (A) Fluorescence spectra of HPTS inside $C_{18:1}$ vesicles before acid bath (black line), after acid bath within 1 min (red line) and the addition of triton X-100 (bule line). (B) The lowly and highly-magnified CLSM (confocal laser scanning microscope) images of $C_{18:1}$ vesicles after acid bath for 48 h, red (TRITC), green (HPTS).

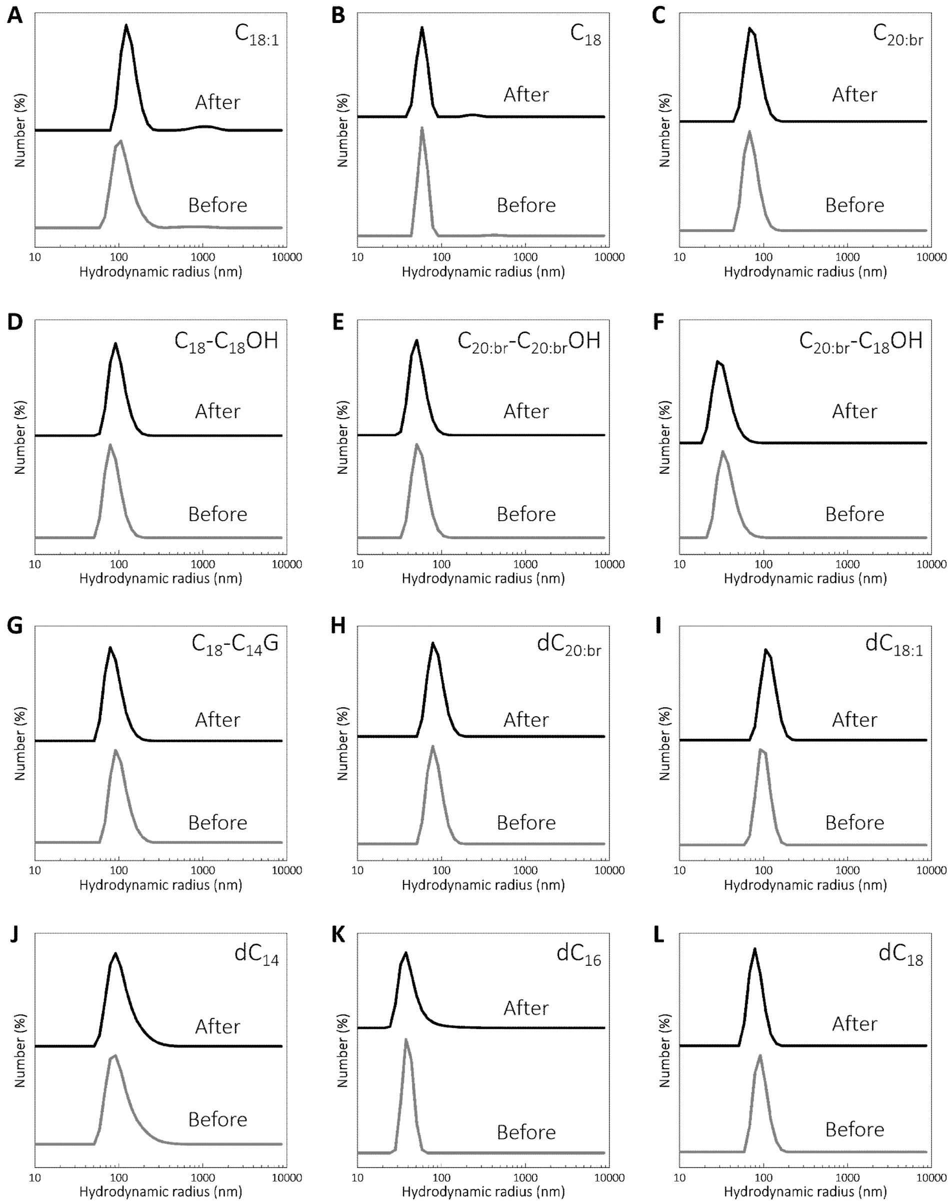

Figure S4. Hydrodynamic radius of various vesicles before and after acid bath measured by dynamic light scattering. The mole ratio of acid/alcohol is 2:1. (A) $C_{18:1}$; (B) C_{18}; (C) $C_{20:br}$; (D) C_{18}-C_{18}OH; (E) $C_{20:br}$-$C_{20:br}$OH; (F) $C_{20:br}$-C_{18}OH; (G) C_{18}-C_{14}G; (H) d$C_{20:br}$; (I) d$C_{18:1}$; (J) dC_{14}; (K) dC_{16}; (L) dC_{18}.

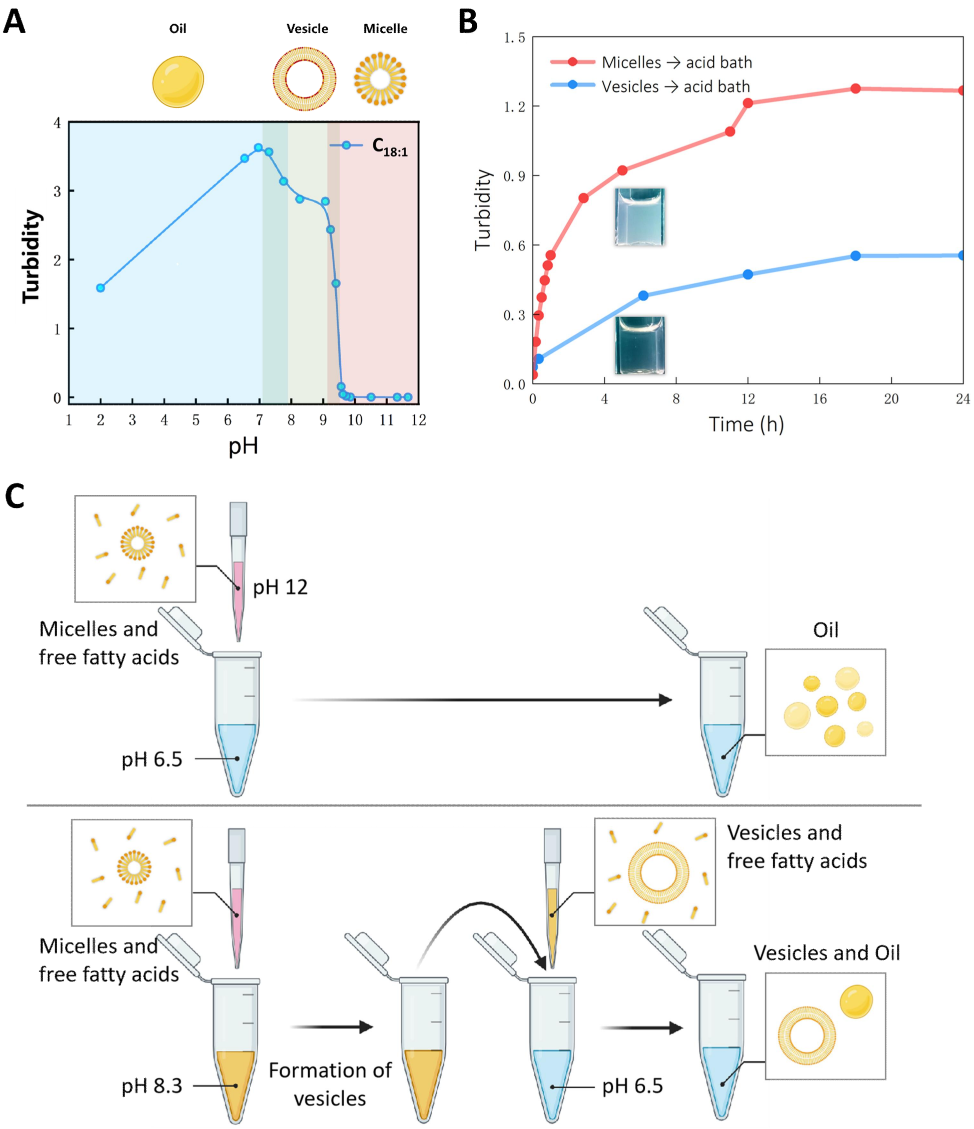

Figure S5. Resistance of fatty acid vesicles to pH changes. (A) Turbidity titration curve of oleic acid ($C_{18:1}$). (B) The turbidity of oleic acid micelles and preformed vesicles after acid bath (pH 6.5) over time. (C) Schematic diagram of comparison between fatty acid ($C_{18:1}$) micelles and preformed vesicles after acid bath.

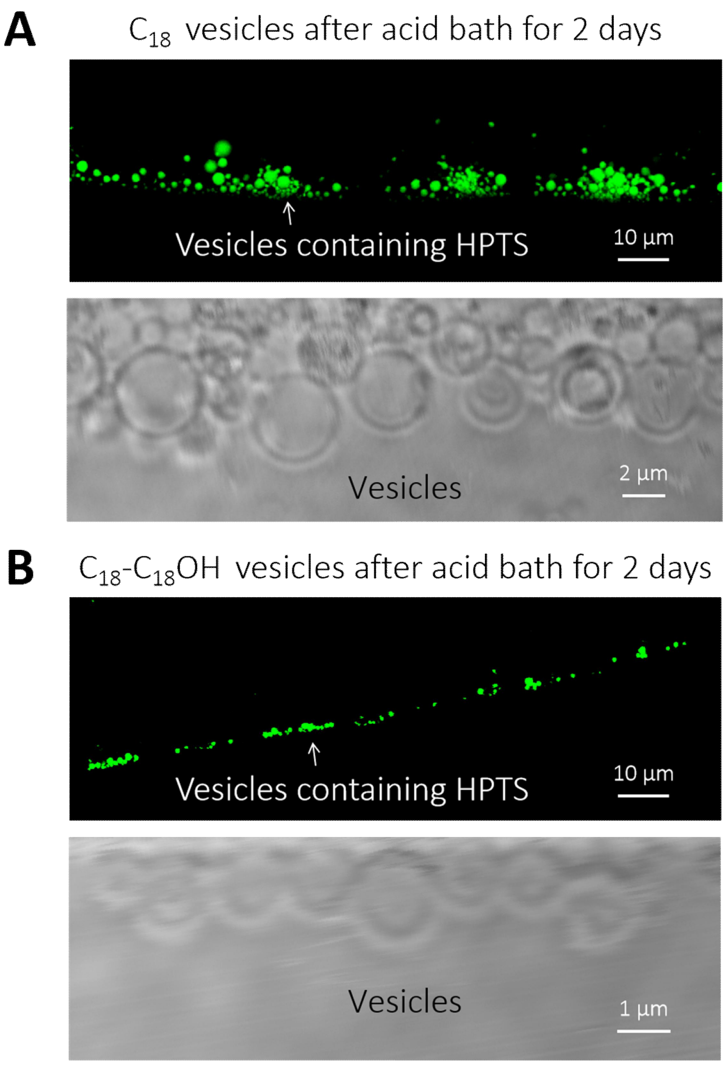

Figure S6. The resistance of C_{18} or C_{18}-C_{18}OH vesicles to acid bath. (A) The CLSM images of C_{18} vesicles after acid bath (pH 6.5) for 2 days. Upper panel, vesicles containing HPTS (green). Lower panel, highly-magnified bright field. (B) The CLSM images of C_{18}OH vesicles after acid bath for 2 days. The mole ratio of acid/alcohol is 2:1. Upper panel, vesicles containing HPTS (green). Lower panel, highly-magnified bright field.

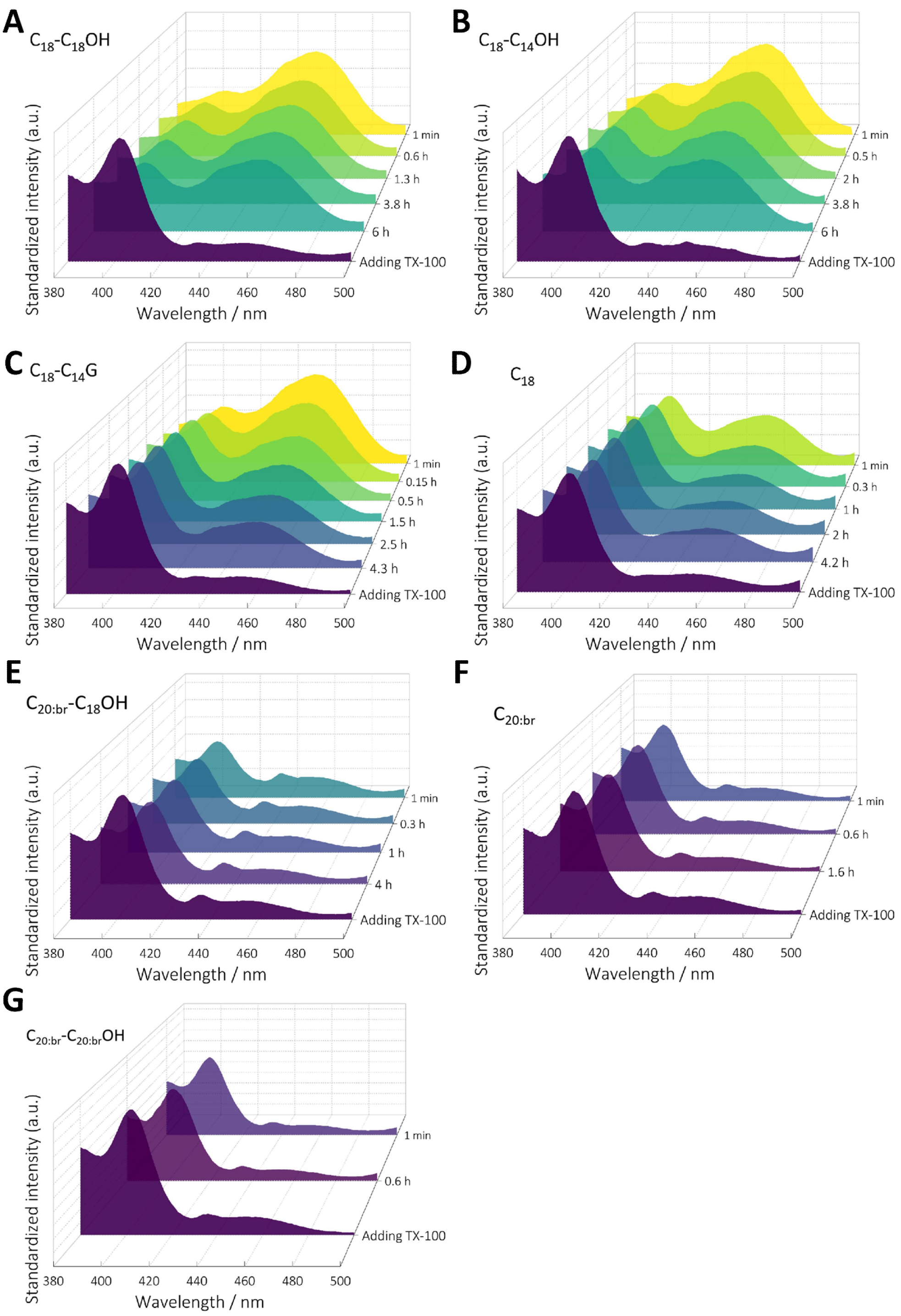

Figure S7. Excitation spectra of HPTS inside vesicles composed of fatty acids and their derivatives after acid bath over time. After adding triton X-100 (TX-100) to break vesicles, the pH outside the vesicles was obtained. (A) C_{18}-$C_{18}OH$; (B) C_{18}-$C_{14}OH$; (C) C_{18}-$C_{14}G$; (D) C_{18}; (E) $C_{20:br}$-$C_{18}OH$; (F) $C_{20:br}$; (G) $C_{20:br}$-$C_{20:br}OH$.

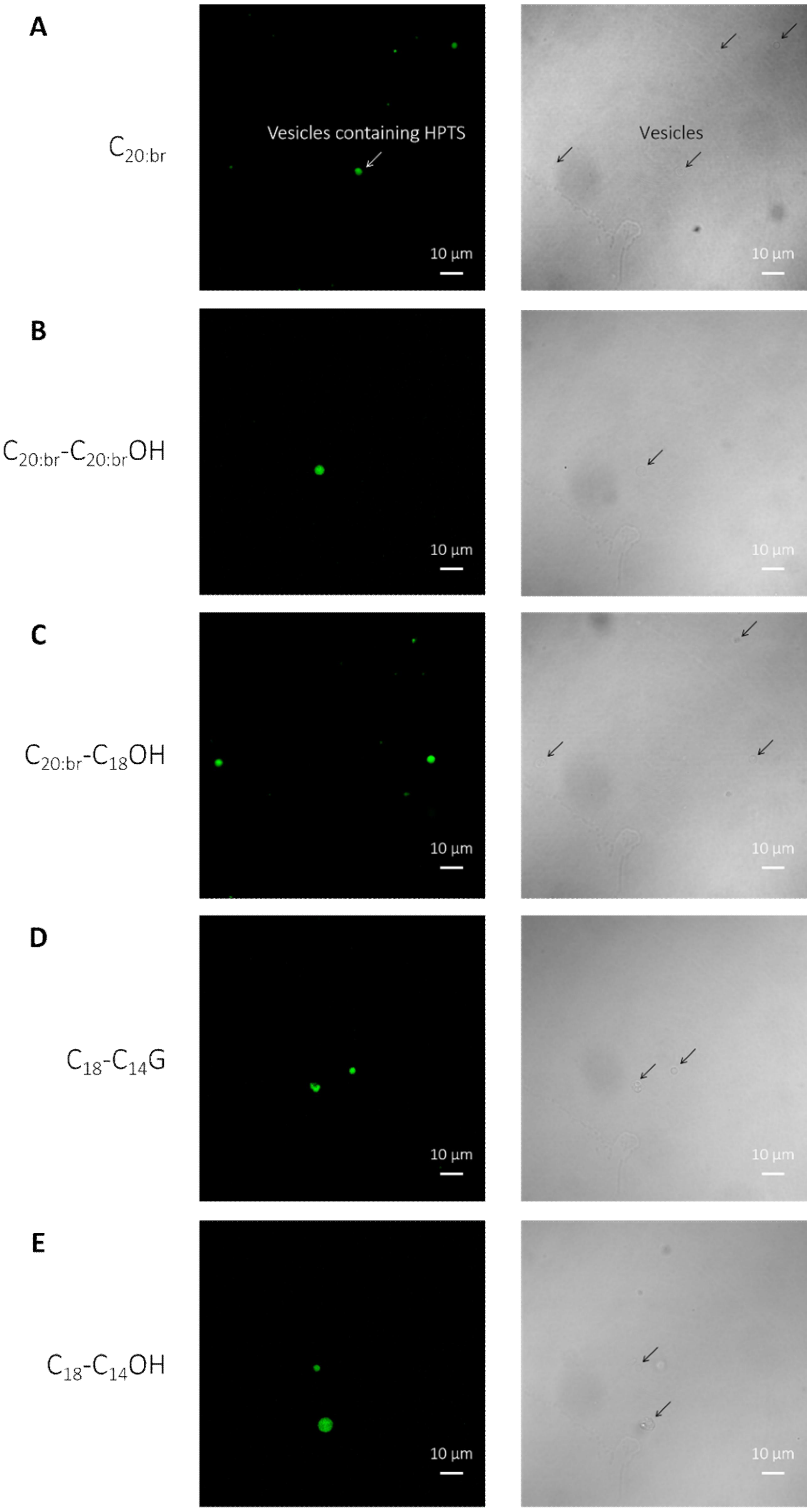

Figure S8. The resistance of other fatty acid vesicles to acid bath. The CLSM images of (A) $C_{20:br}$, (B) $C_{20:br}$-$C_{20:br}OH$, (C) $C_{20:br}$-$C_{18}OH$, (D) C_{18}-$C_{14}G$ or (E) C_{18}-$C_{14}OH$ vesicles after acid bath (pH 6.5) for 4 h. The mole ratio of acid/alcohol was 2:1. Left panel, vesicles containing HPTS (green). Right panel, bright field. The arrow marks the vesicles.

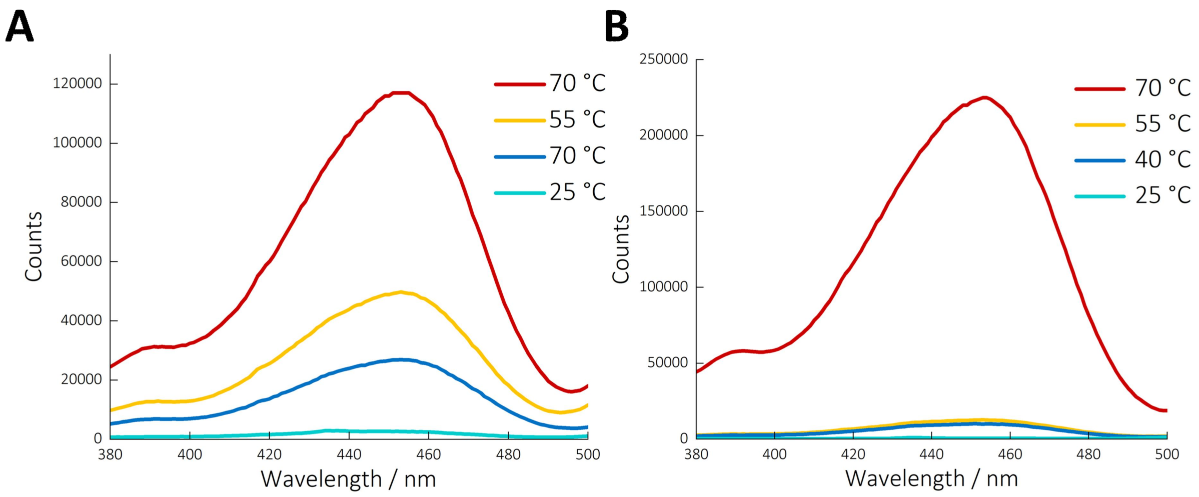

Figure S9. The relationship between the formation of vesicles and temperature. (A) Excitation spectra of HPTS inside C_{18}-$C_{18}OH$ vesicles prepared at different temperatures. (B) Excitation spectra of HPTS inside C_{18} vesicles prepared at different temperatures.

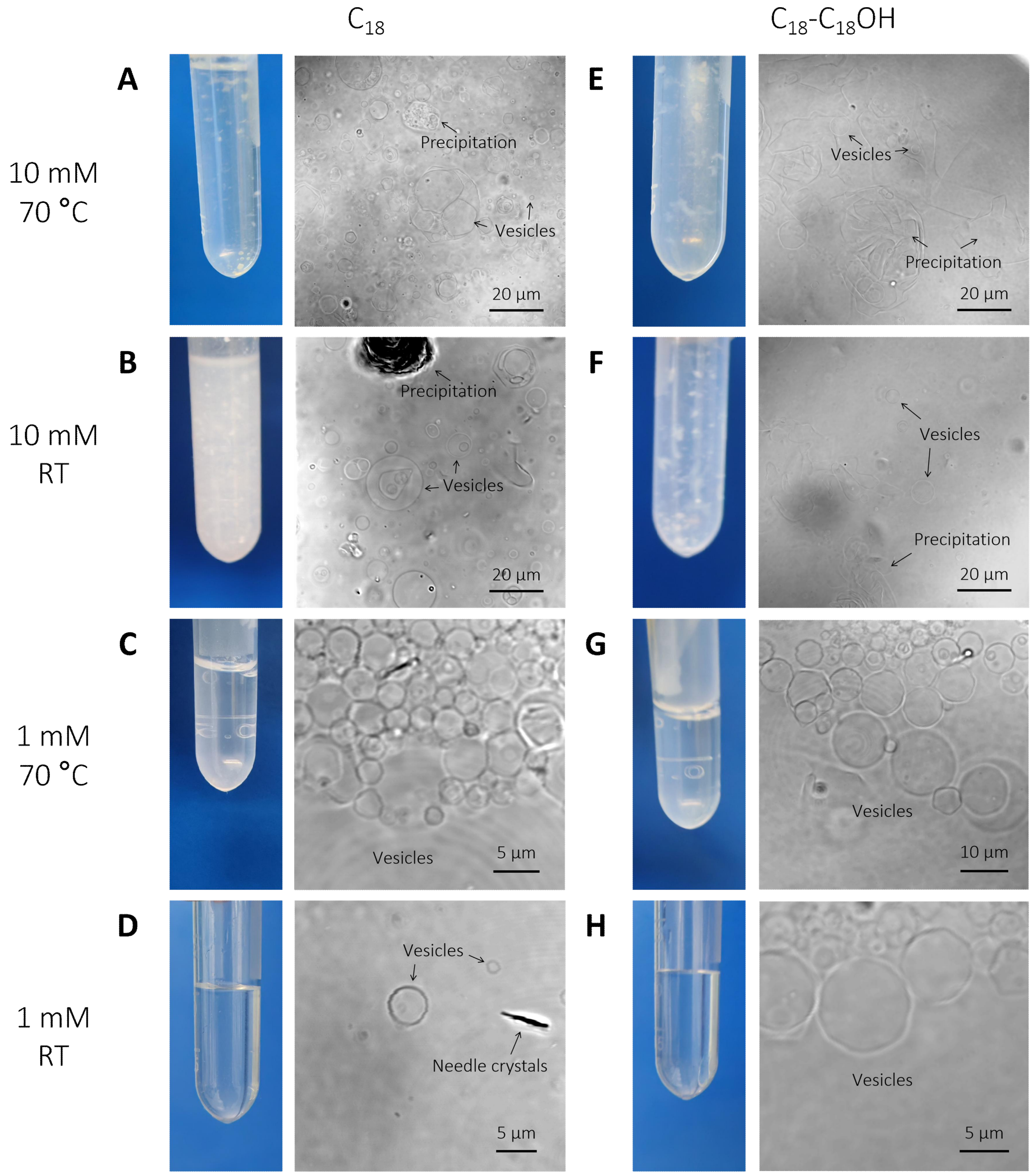

Figure S10. The influence of temperature and concentration on vesicles. Photos and microscopy images of C_{18} (A-D) or C_{18}-$C_{18}OH$ (E-H) under different conditions. The molar ratio $C_{18}/C_{18}OH$ is 2:1. (A, E) When total concentration is 10 mM at 70 °C, the solution was relatively clear and transparent. Numerous vesicles and amorphous precipitation were observed by microscopy imaging. (B, F) After cooling down to room temperature (~20 °C) for 2 days, the C_{18} solution became turbid and more precipitation appeared, but numerous vesicles were still present. The C_{18}-$C_{18}OH$ solution was relatively clear and transparent. Numerous vesicles of C_{18}-$C_{18}OH$ were observed. (C, D, G, H) When total concentration was 1 mM at 70 °C or after cooling down to room temperature (~20 °C) for 2 days, the solution of C_{18} or C_{18}-$C_{18}OH$ was relatively clear and transparent. Numerous vesicles and some needle crystals were observed.

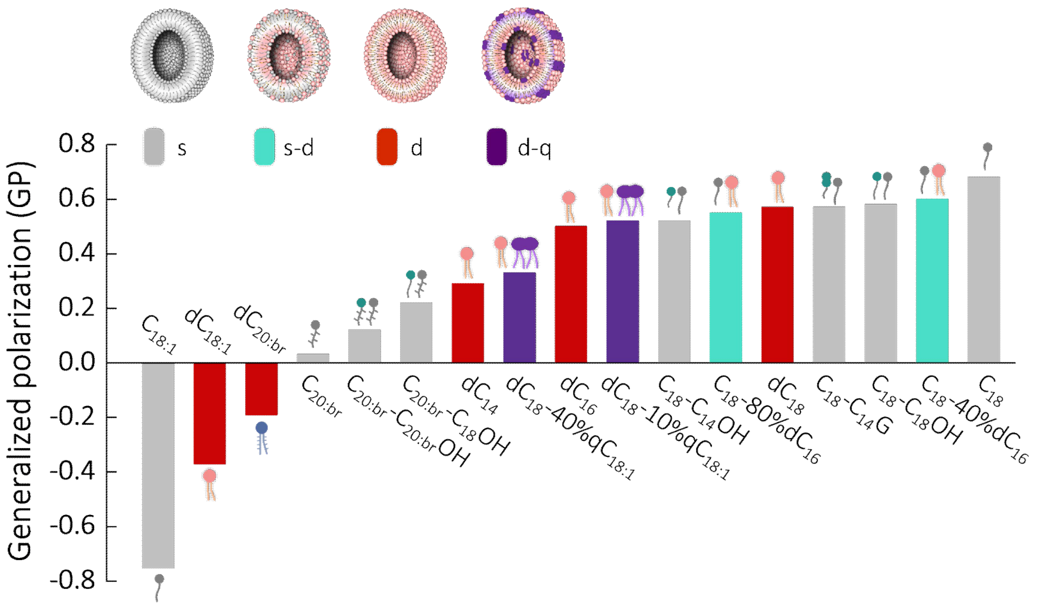

Figure S11. The GP value of vesicles assembled with fatty acids/alcohols or phospholipids at room temperature (~20 °C). The mole ratio of acid/alcohol is 2:1. s, single-chain (grey); s-d, single-chain and double-chain (cyan); d, double-chain (red); d-q, double-chain and quadruple-chain (purple).

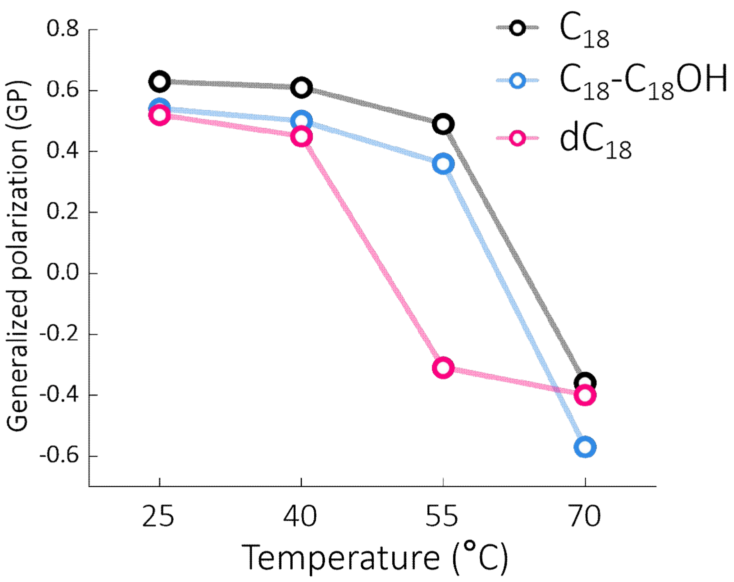

Fig. S12 The influence of temperature on membrane fluidity. The GP value of C_{18}, C_{18}-$C_{18}OH$ and dC_{18} vesicles at 25 °C, 40 °C, 55 °C and 70 °C, respectively.

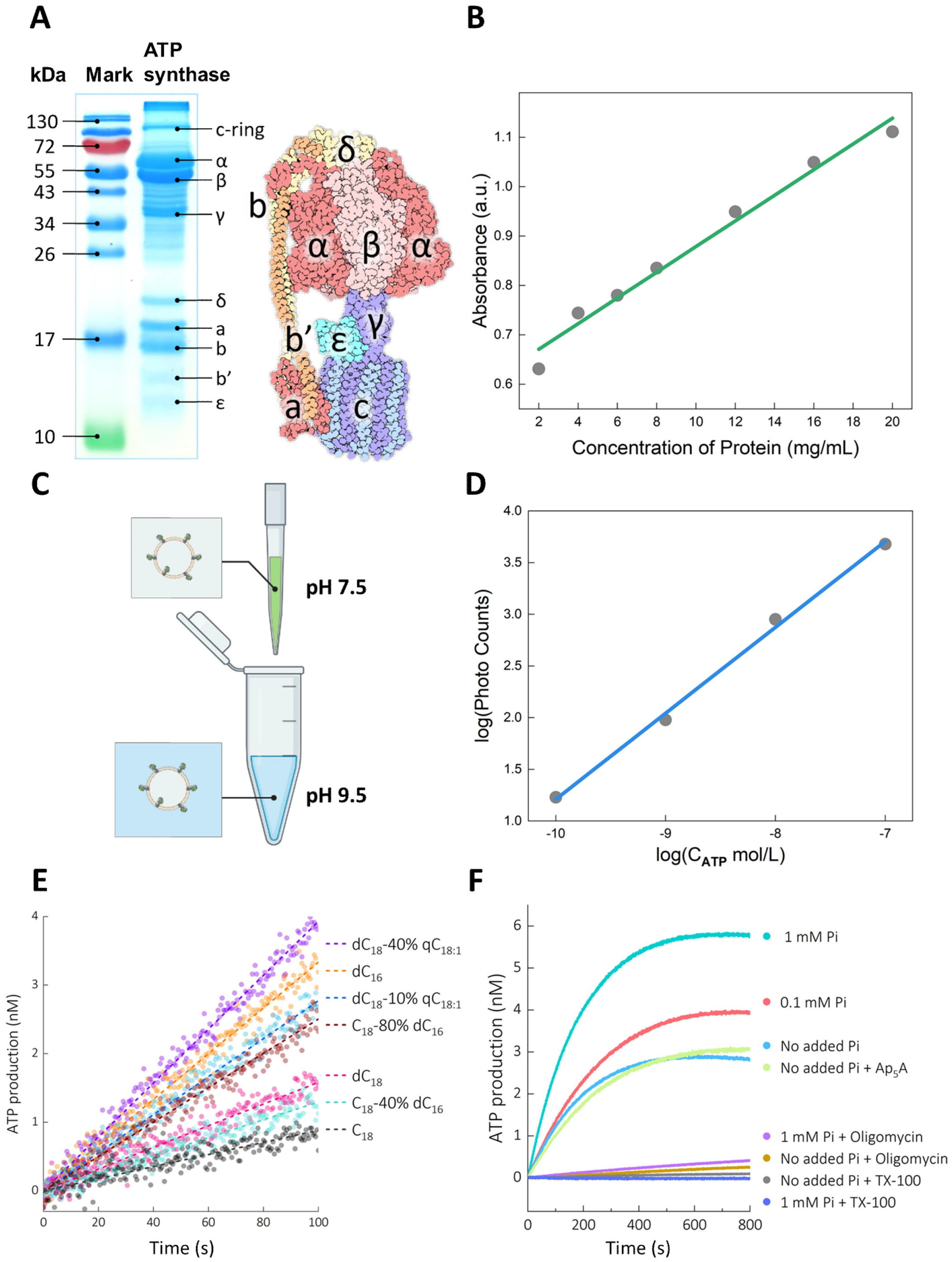

Figure S13. Verification of structure and function of ATP synthase. (A) The SDS-PAGE image of the extracted ATP synthase. (B) Standard curve used to determine concentration of ATPase synthase. (C) Schematic diagram of proton gradient formation by base bath. (D) Standard curve used to determine concentration of ATP. (E) ATP production of ATP synthase-reconstituted vesicles over time for calculating the relative apparent ATP synthesis rate based on the slope of the initial 100 seconds. (F) ATP production of ATP synthase-reconstituted C_{18} vesicles over time. ATP synthesis is phosphate-dependent. When no additional phosphate (Pi) is added, ATP synthesis also occurred, because of the residual phosphate buffer in the ATP synthase extraction solution. Oligomycin is an ATP synthase inhibitor. TX-100 can break the vesicles. Ap5A is an adenylate kinase inhibitor.

Supplemental References

1. Jordan, S. F., Rammu, H., Zheludev, I. N., Hartley, A. M., Maréchal, A. and Lane, N. (2019). Promotion of protocell self-assembly from mixed amphiphiles at the origin of life. Nat. Ecol. Evol. *3*, 1705-1714. https://doi.org/10.1038/s41559-019-1015-y
2. Budin, I., Debnath, A. and Szostak, J. W. (2012). Concentration-Driven Growth of Model Protocell Membranes. J. Am. Chem. Soc. *134*, 20812-20819. https://doi.org/10.1021/ja310382d
3. Li, Z., Yu, F., Xu, X., Wang, T., Fei, J., Hao, J. and Li, J. (2023). Photozyme-catalyzed ATP generation based on ATP synthase-reconstituted nanoarchitectonics. J. Am. Chem. Soc. *145*, 20907-20912. https://doi.org/10.1021/jacs.3c06090
4. Dezi, M., Di Cicco, A., Bassereau, P. and Lévy, D. (2013). Detergent-mediated incorporation of transmembrane proteins in giant unilamellar vesicles with controlled physiological contents. Proc. Natl. Acad. Sci. U. S. A. *110*, 7276-7281. https://doi.org/10.1073/pnas.1303857110
