# Supplementary material for: Chemiosmotic ATP synthesis by minimal protocells
Source: Cell Rep Phys Sci. 2025 Mar 19;6(3):102461. doi: 10.1016/j.xcrp.2025.102461 (PMC11922820; doi:10.1016/j.xcrp.2025.102461)
Supplement: Document S2. Article plus supplemental information [file mmc2.pdf]

Cell Reports
Physical Science

Report

# Chemiosmotic ATP synthesis by minimal protocells

## Graphical abstract

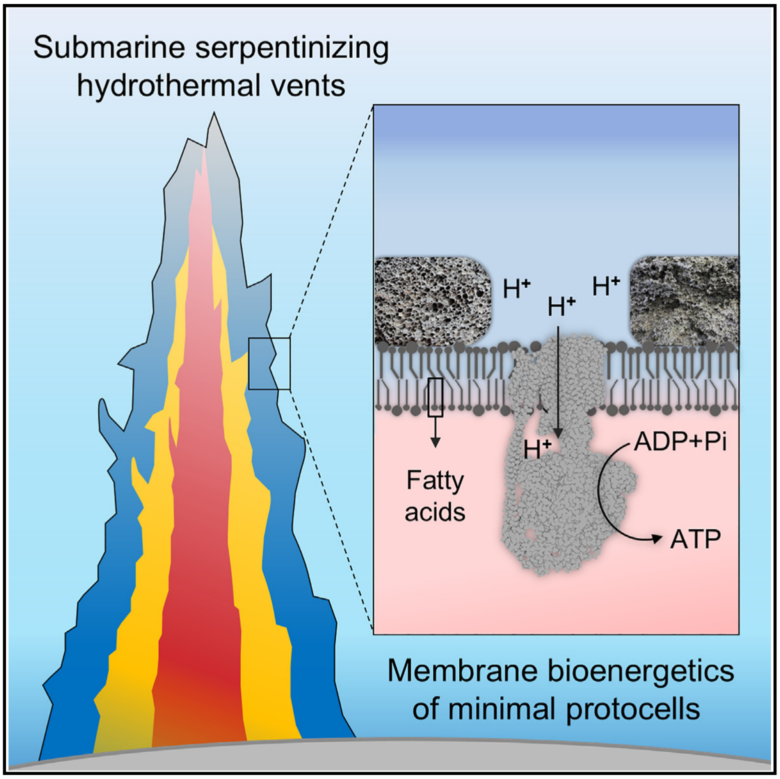


## Highlights

- Fatty acid membranes can maintain sufficient proton gradients
- ATP synthase-embedded minimal protocells produce ATP
- The protocell membranes have an influence on ATP synthesis

## Authors

Fanchen Yu, Jinbo Fei, Yi Jia,
Tonghui Wang, William F. Martin,
Junbai Li

## Correspondence

jbli@iccas.ac.cn

## In brief

Yu et al. report that fatty acid membranes can maintain sufficient proton gradients, which drive ATP synthase to produce ATP. The chemiosmotic ATP synthesis by these minimal protocells sheds substantial light on early evolutionary intermediates of membrane bioenergetics.

Yu et al., 2025, Cell Reports Physical Science 6, 102461
March 19, 2025 © 2025 The Author(s). Published by Elsevier Inc.
https://doi.org/10.1016/j.xcrp.2025.102461

Cell Reports
Physical Science

CellPress
OPEN ACCESS

Report

# Chemiosmotic ATP synthesis by minimal protocells

Fanchen Yu,[1,3] Jinbo Fei,[1,3] Yi Jia,[1] Tonghui Wang,[1,3] William F. Martin,[2] and Junbai Li[1,3,4,*]
[1]Beijing National Laboratory for Molecular Sciences (BNLMS), CAS Key Lab of Colloid, Interface and Chemical Thermodynamics, Institute of Chemistry, Chinese Academy of Science, Beijing 100190, China
[2]Institute of Molecular Evolution, University of Düsseldorf, 40225 Düsseldorf, Germany
[3]University of Chinese Academy of Sciences, Beijing 100049, China
[4]Lead contact
*Correspondence: jbli@iccas.ac.cn
https://doi.org/10.1016/j.xcrp.2025.102461

## SUMMARY

Energy conservation is crucial to life's origin and evolution. The common ancestor of all cells used ATP synthase to convert proton gradients into ATP. However, pumps generating proton gradients and lipids maintaining proton gradients are not universally conserved across all lineages. A solution to this paradox is that ancestral ATP synthase could harness naturally formed geochemical ion gradients with simpler environmentally provided precursors preceding both proton pumps and biogenic membranes. This runs counter to traditional views that phospholipid bilayers are required to maintain proton gradients. Here, we show that fatty acid membranes can maintain sufficient proton gradients to synthesize ATP by ATP synthase under the steep pH and temperature gradients observed in hydrothermal vent systems. These findings shed substantial light on early membrane bioenergetics, uncovering a functional intermediate in the evolution of chemiosmotic ATP synthesis during protocellular stages postdating the ATP synthase's origin but preceding the advent of enzymatically synthesized cell membranes.

## INTRODUCTION

Experimental evidence for the processes of energy conservation in the first cells on Earth is scarce, but top-down comparative studies[1–4] combined with the bottom-up construction of bio-like nanoarchitectures[5–11] render the problem tractable. Though ATP is the universal energy currency for virtually all biochemical or cellular activities that require energy,[12,13] its synthesis is afforded by a single molecular species, the ATP synthase, which converts ADP and phosphate into ATP using proton-motive force across phospholipid membranes.[7,14–19] The ATP synthase is as universally conserved as ribosomes and genetic code, while proton pumps that generate the ion gradients it requires are not.[20] This suggests that the ATP synthase appeared before the last universal common ancestor (LUCA) of all cells diverged into bacteria and archaea[4,20–22] (Scheme 1A).

Bacteria and archaea are located at roots of the Tree of Life,[23,24] and their ATP synthase is conserved. But these two prokaryotic domains have distinct membrane molecules structures[20,25] and lipid synthesis pathways.[26,27] Bacterial phospholipid tails are straight-chain fatty acids (mainly 18 carbons), while archaeal tail chains are branched-chain isoprenoids (mainly $C_{20}$ phytanyl chains).[28,29] Their unrelated biosynthetic pathways[20,25] suggest that protocells before the LUCA had simpler primitive lipids,[30] like single-chain fatty acids[31–35] or isoprenoid acids,[30,31,36] rather than double-chain phospholipid glycerol conjugates (Scheme 1A). Fatty-acid-based protocells are, however, thought to be unable to support the chemiosmotic ATP synthesis[37,38] because primitive membranes assembled from short-chain or unsaturated fatty acids have high membrane fluidity and are "leaky"; that is, they are permeable to small molecules[33] and/or protons[39] and, hence, unable to maintain stable ion gradients.

Serpentinizing hydrothermal systems provide an environment highly conducive to chemiosmotic energy conservation.[20,40] Since there was first water on Earth, serpentinizing hydrothermal vents have continuously forced warm (40°C–100°C)[41–44] alkaline water (pH = 9–11)[45] to interface with ocean water (pH = 6.5–7),[46] generating stable, natural, geochemical proton and temperature gradients[40,47–49] (Scheme 1A). These proton gradients could, in principle, serve as the evolutionary precursor of biological proton pumps. However, this requires that protocells with abiotically primitive lipid membranes could harness such geochemically formed pH gradients.[4] Heat flux generated by temperature gradients of hydrothermal vents contributes to thermophoretic enrichment and the assembly of amino acids, nucleotides, and, importantly, lipids.[50] Yet, the ATP synthase requires a proton-tight membrane of hydrophobic molecules with the thickness of an ATP synthase membrane subunit to function.[7,51,52] Fatty acids up to 18 carbons are synthesized from $H_2$ and $CO_2$ with simple metal catalysts under conditions of hydrothermal vents, providing a source of primordial lipid monomers.[53–57] However, the crucial question of whether membranes consisting of such simple, abiotically formed, straight-chain lipids can support ATP synthase function has not been experimentally answered to date.

Here, we show that membranes consisting solely of solitary long-chain saturated fatty acids maintain proton gradients that


This is an open access article under the CC BY license (http://creativecommons.org/licenses/by/4.0/).

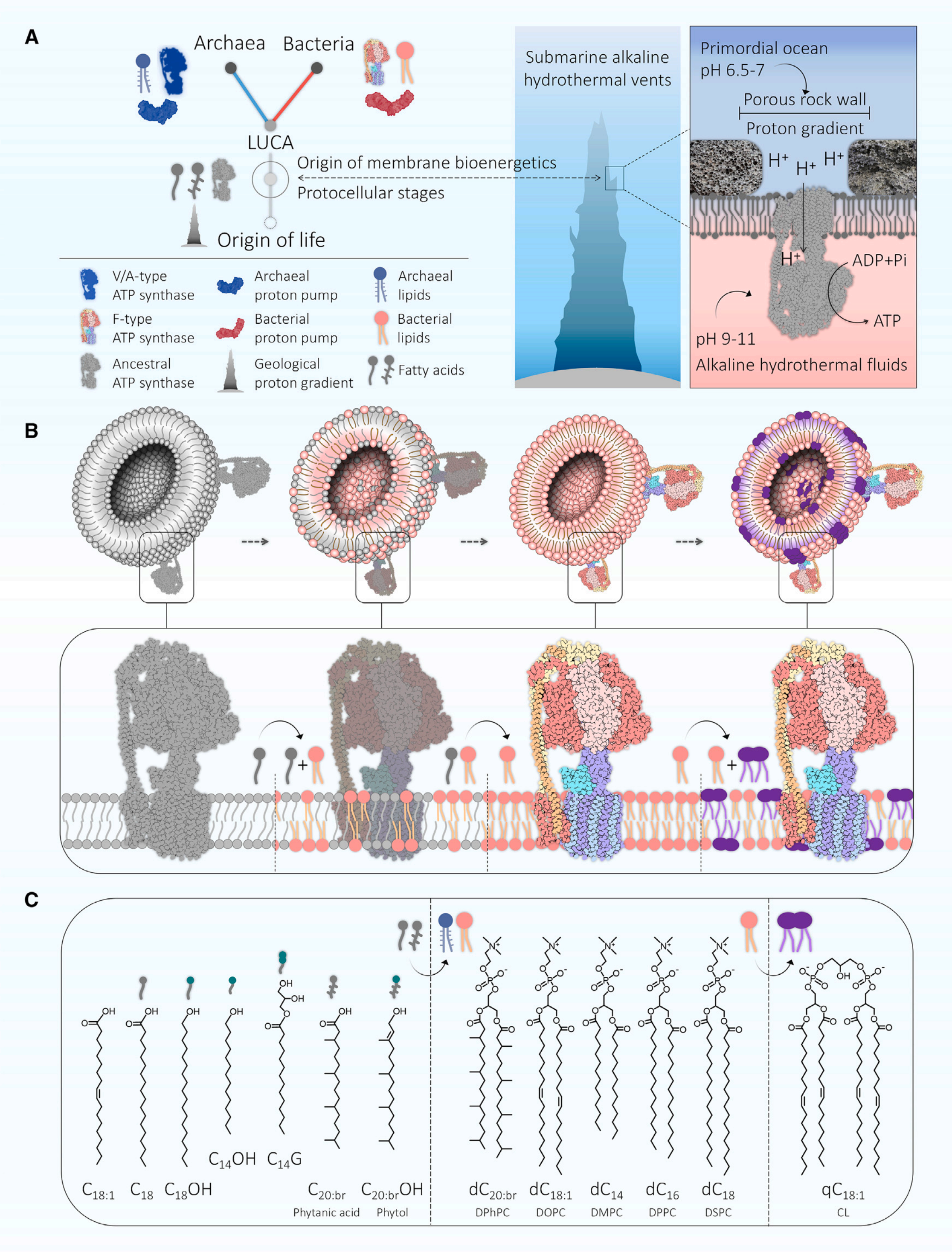


**Scheme 1. The possible evolution of membrane bioenergetics and its conceptual model protocells**

(A) The evolutionary relationship of bacteria to archaea suggests that prior to the last universal common ancestor (LUCA), early life went through a protocellular stage with ATP synthase and fatty acid membranes but without proton pumps. In this stage, the ATP synthase could have been driven by geochemical proton gradients across the interface between the primordial oceans (pH = 6.5–7) and the alkaline hydrothermal fluid (pH = 9–11) of serpentinizing hydrothermal vents.

*(legend continued on next page)*

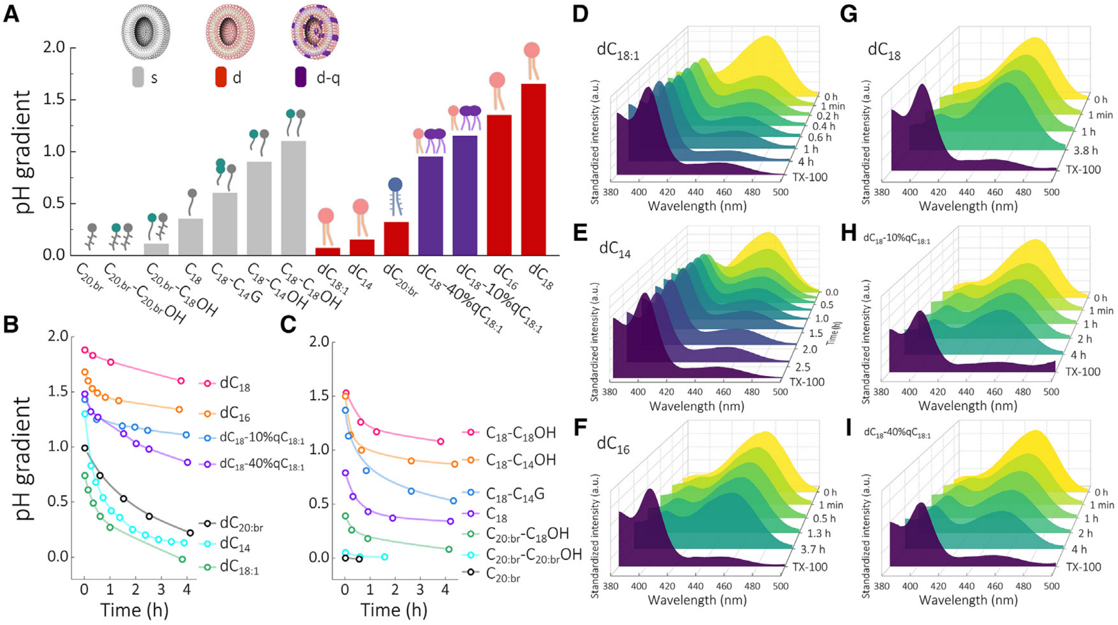


**Figure 1. Proton permeability of membranes assembled with fatty acids or phospholipids**

(A) pH gradients of vesicles after acid bath for 3 h at room temperature (RT; ~20°C). The molar ratio of fatty acid/alcohol is 2:1. s, single chain (gray); d, double chain (red); d-q, double chain and quadruple chain (purple).

(B and C) pH gradients of vesicles composed of phospholipids (B) or fatty acids and their derivatives (C) over time at RT. Their first point of curves starts at 1 min after an acid bath, considering that the pH jump occurs within 1 min, due to residual HPTS outside vesicles and electrically uncompensated proton influx.[61,62] Next, there are two phases of fast and slow pH decay due to the transient-pore mechanism, solubility-diffusion mechanism, and counterion flux limitation.[63]

(D–I) Excitation spectra of HPTS inside vesicles composed of phospholipids after acid bath over time at RT. After adding TX-100 to break vesicles, the pH outside the vesicles was obtained. (D) $dC_{18:1}$, (E) $dC_{14}$, (F) $dC_{16}$, (G) $dC_{18}$, (H) $dC_{18}$-10%$qC_{18:1}$, and (I) $dC_{18}$-40%$qC_{18:1}$.

power an ATP synthase to produce ATP in a minimal protocell (Scheme 1B). Structures of membrane molecules and temperature in the assembled system can modulate membrane assembly, its ability to maintain proton gradients, fluidity and ATP synthesis. The results uncover an evolutionary intermediate in primordial bioenergetics linking ATP synthase function in abiotic fatty acid membranes using geochemically formed gradients to ATP synthesis in biochemically synthesized phospholipid bilayers.

## RESULTS AND DISCUSSION

### Long-chain saturated fatty acid membranes outperform some phospholipid membranes in pH gradient stability

We began by investigating proton gradients. Vesicles containing a pH fluorescent probe (8-hydroxypyrene-1, 3, 6-trisulfonic acid trisodium salt [HPTS])[19] were obtained (Figure S1). According to the hydrothermal vent theory,[1,2,4,40] protocells harnessed natural geological proton gradients for ATP synthesis.[20] Therefore, we formed analogous proton gradients using the acid bath method, changing pH outside the vesicles from about pH 9.5 to 6.5 (Figure S2). Because the chain lengths of phospholipids in modern cell membranes are typically 18 carbons,[58] we mainly chose building blocks with similar chain lengths (Scheme 1C). Consistent with previous studies, long-chain unsaturated oleic acid ($C_{18:1}$) vesicles alone cannot maintain a proton gradient[39]; their spectra coincide within 1 min after an acid bath and after the addition of detergent TX-100, breaking the vesicles (Figure S3A). A confocal laser scanning microscope (Figure S3B) and dynamic light scattering (Figure S4) show that the acid bath did not directly break $C_{18:1}$ vesicles (Figure S5).

Next, we tested long-chain saturated fatty acid ($C_{18}$) vesicles as a reference for subsequent comparisons. Numerous $C_{18}$ vesicles were still observed after spending 2 days in an acid bath (Figure S6). We found that simple $C_{18}$ vesicles maintain a pH gradient over 0.35 pH units after a 3 h acid bath (Figures 1A and S7), a pH gradient that is within the range of 0.3–0.5 pH units measured for growing *E. coli*.[59,60] In contrast, the pH gradient of unsaturated double-chain phospholipid vesicles with the same chain length ($dC_{18:1}$) approaches 0 after 3 h, but its pH gradient did not dissipate within 1 min like $C_{18:1}$ vesicles (Figures 1B and 1D). Moreover, the pH gradient of saturated $dC_{18}$ is still

(B) At the onset of membrane bioenergetics, a simple fatty acid membrane can maintain proton gradients to drive the ATP synthesis via the ATP synthase. Subsequent adaptation to the free-living lifestyle fosters the transition from single-chain fatty acids to double-chain and quadruple-chain phospholipids.

(C) The structural formula of building blocks. $C_{18:1}$, oleic acid; $C_{18}$, stearic acid; $C_{18}$OH, stearyl alcohol; $C_{14}$OH, myristyl alcohol; $C_{14}$G, 1-monomyristoyl glycerol; $C_{20:br}$, phytanic acid; $C_{20:br}$OH, phytol; $dC_{20:br}$, DPhPC; $dC_{18:1}$, DOPC; $dC_{14}$, DMPC; $dC_{16}$, DPPC; $dC_{18}$, DSPC; $qC_{18:1}$, CL, cardiolipin.

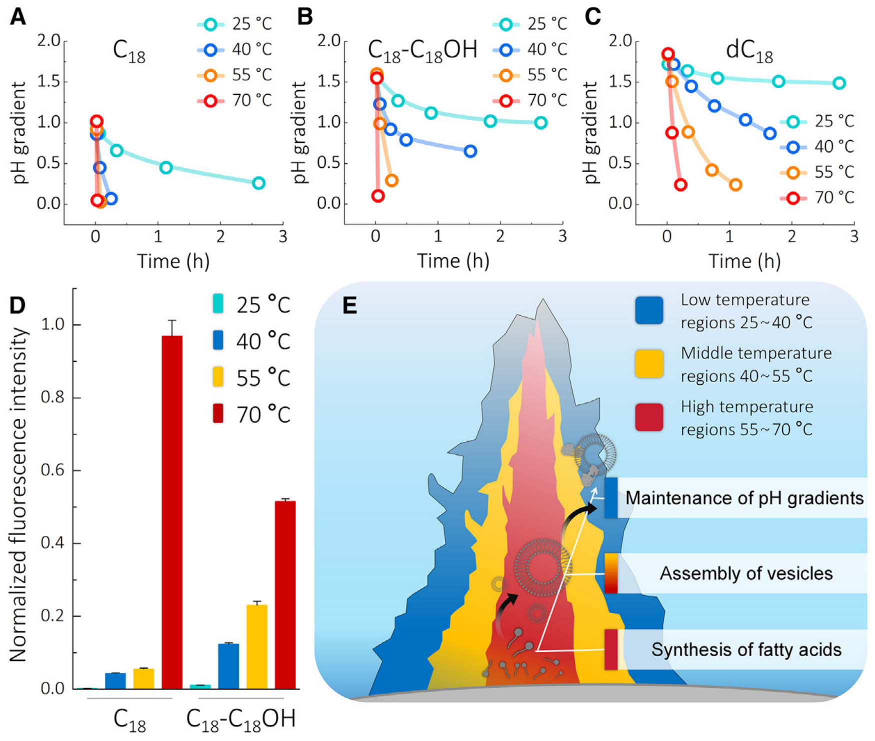


**Figure 2. The effect of temperature on the proton gradients and formation of protocells**

(A–C) The pH gradients of (A) $C_{18}$, (B) $C_{18}$-$C_{18}OH$, and (C) $dC_{18}$ vesicles over time at different temperatures: 25°C (cyan), 40°C (blue), 55°C (yellow), and 70°C (red).

(D) Fluorescence intensity at 460 nm of $C_{18}$ and $C_{18}$-$C_{18}OH$ vesicles prepared at different temperatures: 25°C (cyan), 40°C (blue), 55°C (yellow), and 70°C (red). Data are represented as mean ± SEM.

(E) Scheme of the roles of temperature gradients in alkaline hydrothermal vents for protocells. High-temperature regions contribute to the synthesis of fatty acids and the formation of vesicles, while relatively low-temperature regions allow maintaining proton gradients.

over 1.6 pH units, approximately 5 times that of $C_{18}$ vesicles (Figures 1A and 1G). These findings show that doubling the chain number or increasing the degree of saturation improves the ability of protocell fatty acid membranes to maintain proton gradients.

The presence of two hydrophobic chains linked to glycerol per lipid monomer is a strictly conserved feature of both bacterial and archaeal membranes[25–29,31] and, hence, an important evolutionary advance from protocell-type to enzymatically synthesized membranes. To investigate the effect of further doubling the chain number, we tested a representative cardiolipin ($qC_{18:1}$). $qC_{18:1}$ has four unsaturated tail chains, located in the mitochondrial inner membrane and in some bacteria, where its content reaches up to 10%–20%.[64] The results show that increasing the molar ratio of $qC_{18:1}$ in $dC_{18}$ vesicles increases proton permeability (Figure 1A), while the pH gradients are still higher compared to those in $C_{18}$ and $dC_{18:1}$ vesicles. Cardiolipin is not known to be conducive to maintaining proton gradients but exerts regulatory roles instead.[64]

To explore the interval of proton permeability required to maintain a proton gradient capable of energy conversion in protocells, we tested phospholipids with shorter chain lengths. When the phospholipid chain length decreases to 14 carbons ($dC_{14}$), the ability to maintain a proton gradient is lower than in $C_{18}$ vesicles (Figure 1A). Reconstituted ATP synthase in $dC_{14}$ glycerol ester phospholipid vesicles has previous been studied.[7,14–19]

Mixing fatty alcohols into fatty acid vesicles can effectively enhance membrane stability[31,65] and enhance proton gradient maintenance (Figure 1). Increasing the chain length of fatty alcohols can also decrease membrane permeability. Membrane permeability of fatty acid glycerides is greater than that of fatty alcohols, likely due to the larger glycerol head group, which decreases membrane tightness.

Our experiments so far have employed bacterial-type aliphatic chains as hydrophobic components. To probe the ability of archaeal-type hydrophobic tails, we generated vesicles composed of branched-chain fatty acids. They failed, however, to maintain the proton gradients, although the vesicles themselves were still observed after acid bath treatment (Figures 1 and S8). Their pH gradients dissipated within 1 min. It is more likely that archaeal-type fatty acids are produced by biological synthesis at archaeal stages rather than by abiotic processes.[25–29,31] Our results indicate that protocell membranes composed of abiotically synthesized straight-chain fatty acids can maintain proton gradients. The fatty acids, synthesized from $H_2$ and $CO_2$ in serpentinizing systems,[53–56] could have served to maintain geochemical ion gradients in the common ancestor of archaea and bacteria.[4,20]

## Protocell membrane bioenergetics require temperature gradients

Temperature affects the formation of long-chain saturated fatty acid vesicles[66] and their membrane permeability. $C_{18}$ vesicles at 40°C, $C_{18}$-$C_{18}OH$ vesicles at 55°C, and $dC_{18}$ vesicles at 70°C lose proton gradients within 15 min (Figures 2A–2C). However, vesicle formation is improved at 70°C, and the numbers of obtained vesicles containing fluorescence pH probes decreases sharply with decreasing temperature (Figures 2D and S9). The precipitation of $C_{18}$ or $C_{18}$-$C_{18}OH$ was not significant at 1 mM, and numerous vesicles were still observed after cooling down from 70°C to room temperature (RT) (Figure S10).

While the formation of long-chain saturated fatty acid vesicles requires higher temperatures (70°C), maintaining proton gradients requires lower temperatures (40°C). The requirement of different temperature ranges for thermophoretic concentration mechanisms,[50] vesicle formation, and ion gradient maintenance implicate an environment with temperature gradients as the site

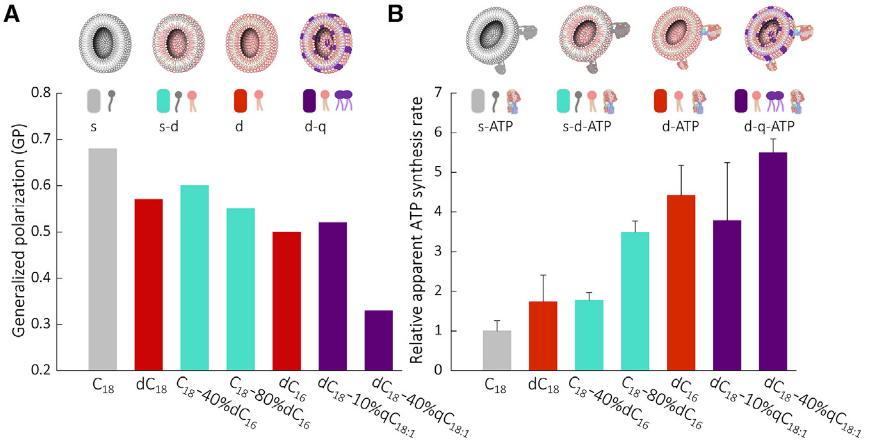


**Figure 3. ATP bioenergy synthesis of model protocells reconstituted with ATP synthase**

(A) Fluidity of membranes assembled with fatty acids or phospholipids. The GP value of vesicles at room temperature (RT; ∼20°C). s, single chain (gray); s-d, single chain and double chain (cyan); d, double chain (red); d-q, double chain and quadruple chain (purple).

(B) Relative apparent ATP synthesis rate of vesicles reconstituted with ATP synthase after base bath at RT. The rate is calculated based on the slope of the initial 100 s of ATP production over time, using the rate of $C_{18}$ as the reference. s-ATP, single-chain fatty acids reconstituted with ATP synthase (gray); s-d-ATP, single-chain fatty acids and double-chain phospholipids reconstituted with ATP synthase (cyan); d-ATP, double-chain phospholipids reconstituted with ATP synthase (red); d-q-ATP, double-chain and quadruple-chain phospholipids reconstituted with ATP synthase (purple). Data are represented as mean ± SEM.

of bioenergetic origin.[67] The naturally existing temperature gradients (40°C–75°C)[41] in vents of serpentinizing hydrothermal systems[68] satisfy the temperature, ion gradient, and lipid monomer synthesis conditions required for ATP synthase function (Figure 2E).

The temperature of primordial ocean is still discussed. Some argue that primordial ocean reached up to 70°C 3.5 billion years ago,[42] while recent findings suggest that primordial oceans had more mild temperatures.[44] Our results show that both fatty acid and phospholipid vesicles fail to maintain proton gradients at high temperatures, which would preclude their function in chemiosmotic ATP synthesis. This suggests that prior to the origin of enzymatic lipid synthesis, the transition from soluble energy-conserving reactions to energy conservation with an ATP synthase[4,20] required a mild temperature range.

## Protocells reveal a trade-off between membrane fluidity and proton permeability for improving ATP synthesis

Membrane fluidity is another key factor impacting membrane protein function.[69–71] We tested the membrane fluidity of vesicles using a fluorescence probe (Laurdan).[72,73] A lower generalized polarization (GP) value indicates higher membrane fluidity. Usually, the GP value of normal cell membranes is about 0.2–0.8.[72] Membrane fluidity increases with the decreasing chain length, and the introduction of $qC_{18:1}$ has a similar affect (Figures 3A and S11). Usually, lower membrane fluidity means lower permeability (Figures 1 and 3A), but when comparing $C_{18}$ with $dC_{18}$, doubling the chain number can simultaneously improve membrane fluidity and the ability to maintain proton gradients.

Generally, when the GP value falls below 0.3, the membranes are in a liquid state.[74] The fatty acid vesicles ($C_{18:1}$, $C_{20:br}$, and $C_{20:br}$-$C_{20:br}$OH at RT and $C_{18}$ and $C_{18}$-$C_{18}$OH at 70°C) at a liquid state cannot maintain proton gradients, while the phospholipid vesicles ($dC_{18:1}$, $dC_{20:br}$, and $dC_{14}$ at RT and $dC_{18}$ at 55°C) at a liquid state can do those (Figures 1, 2, S11, and S12).

We examined protocell membranes for their ability to support and modulate ATP synthesis using reconstituted ATP synthase (Figures 3B and S13). The results show that ATP synthase is functional in fatty acid membranes. The ATP synthesis rate increases with decreasing chain length and increasing chain number. Increasing the molar ratio of phospholipid ($dC_{16}$) in fatty acid ($C_{18}$) vesicles or cardiolipin ($qC_{18:1}$) in phospholipid ($dC_{18}$) vesicles increases the ATP synthesis rate. The ATP synthesis rate for $dC_{16}$ vesicles is 4.4 times compared to that for $C_{18}$ vesicles. Meanwhile, the pH gradient at 3 h for the former is 3.8 times higher than that for the latter. These findings indicate the impact of protocell membrane components on protocell energy metabolism.

The ATP synthesis rate and membrane fluidity show a positive correlation (Figure 3), possibly because lower membrane fluidity imparts greater obstruction to rotary catalysis of ATP synthase, leading to a decrease in the ATP synthesis rate. The increase of chain length in fatty acids maintains higher proton gradients but decreases membrane fluidity in corresponding vesicles (Figures 1A and 3A). This suggests a trade-off in protocell membrane fluidity and proton permeability, limiting the lipid chain length range in protocells that support ATP synthase function, even in the presence of a large proton gradient.

In summary, we have shown that protocells enclosed by simple fatty acid membranes can maintain ion gradients and support ATP synthesis via a rotor-stator ATP synthase. The findings show that interactions between complex proteins and abiotically synthesized fatty acids can support membrane bioenergetics via harnessing natural geological proton gradients generated by serpentinization at hydrothermal vents[1,2,20,40] (Scheme 1A). Observed differences between permissive temperatures for the formation of vesicles and maintenance of proton gradients may indicate that membrane bioenergetics originated in environments with natural gradients[67] rather than in isotropic settings (Figure 2E).

Fatty acid composition in protocell membranes specifies membrane fluidity and proton permeability, properties that influence ATP synthase function, also in modern cells.[51,52,75] The evolutionary transition from abiotically synthesized fatty acid membranes to enzymatically synthesized phospholipids with two chains per monomer improved membrane fluidity and

proton gradient stability to a level that has not been improved in 4 billion years, barring the appearance of tetraether lipids in some thermophilic archaea.[29]

The ATP synthase, one of the most sophisticated proteins known,[75] could function in protocellular lipids before free-living cells arose. This may explain how it is possible that bacteria and archaea share the ATP synthase but independently evolved their biosynthetic pathways for membrane lipids[25]: the primordial ATP synthase might not have required enzymatically synthesized lipids to function. That such a complex protein is so ancient might seem to present a paradox.[76,77] A possible scenario is serpentinizing hydrothermal vents where complex ATP synthase and simple lipids were present before the emergence of free-living cells.[1–4,20–22] Primitive lipids (such as fatty acids) could be synthesized from $H_2$ and $CO_2$ by geological catalysts in serpentinizing hydrothermal vents.[53,55] These primitive lipids self-assembled into membranes for embedding ATP synthase translated by ribosomes.[78] Then, ATP was produced by ATP synthase driven by geological proton gradients of serpentinizing hydrothermal vents as bioenergy currency to fuel the enzymatic synthesis of complex lipids[26,27] and other biochemical activities. Although the catalytic function of individual proteins,[79,80] and even entire enzymatic pathways,[81] can be replaced by inorganic catalysts of serpentinizing hydrothermal systems, the ATP synthase function in protocellular lipids represents a special case: its rotor-stator catalytic mechanism has no inorganic or environmental precursor, while the ion gradient that powers it does. Among the many environments that have been suggested for the origin of biological systems,[47–50,67,68] serpentinizing hydrothermal systems are unique in that they generate natural proton gradients[1–4,20–22] that can power an ATP synthase in primitive fatty acid membranes, connecting Earth chemistry and life chemistry in energy conservation.[20,40]

## METHODS

Details regarding the methods can be found in the supplemental information.

### RESOURCE AVAILABILITY

#### Lead contact

Requests for further information and resources should be directed to and will be fulfilled by the lead contact, Junbai Li (jbli@iccas.ac.cn).

#### Materials availability

This study did not generate new unique reagents.

#### Data and code availability

- All data reported in this paper will be shared by the lead contact upon request.
- This paper does not report original code.
- Any additional information required to reanalyze the data reported in this paper is available from the lead contact upon request.

### ACKNOWLEDGMENTS

This work was funded by the National Natural Science Foundation of China (no. 22193031). W.F.M. acknowledges funding from the European Research Council (no. 10118894).

### AUTHOR CONTRIBUTIONS

Conceptualization, J.L. and F.Y.; supervision, J.L.; methodology, J.L., F.Y., W.F.M., and J.F.; experiments, F.Y. and T.W.; data curation, analysis, and visualization, J.L., F.Y., W.F.M., and J.F.; writing – original draft, F.Y.; writing – review & editing, J.L., W.F.M., Y.J., J.F., and F.Y.

### DECLARATION OF INTERESTS

The authors declare no competing interests.

### SUPPLEMENTAL INFORMATION

Supplemental information can be found online at https://doi.org/10.1016/j.xcrp.2025.102461.

Received: September 20, 2024
Revised: January 17, 2025
Accepted: February 3, 2025
Published: March 4, 2025

### REFERENCES

1. Martin, W.F., Sousa, F.L., and Lane, N. (2014). Energy at life's origin. Science *344*, 1092–1093. https://doi.org/10.1126/science.1251653.
2. Lane, N., and Martin, W.F. (2012). The origin of membrane bioenergetics. Cell *151*, 1406–1416. https://doi.org/10.1016/j.cell.2012.11.050.
3. Baross, J.A. (2018). The rocky road to biomolecules. Nature *564*, 42–43. https://doi.org/10.1038/d41586-018-07262-8.
4. Weiss, M.C., Sousa, F.L., Mrnjavac, N., Neukirchen, S., Roettger, M., Nelson-Sathi, S., and Martin, W.F. (2016). The physiology and habitat of the last universal common ancestor. Nat. Microbiol. *1*, 16116. https://doi.org/10.1038/nmicrobiol.2016.116.
5. Jia, X., Chen, J., Lv, W., Li, H., and Ariga, K. (2023). Engineering dynamic and interactive biomaterials using material nanoarchitectonics for modulation of cellular behaviors. Cell Rep. Phys. Sci. *4*, 101251. https://doi.org/10.1016/j.xcrp.2023.101251.
6. Ariga, K. (2024). Nanoarchitectonics: the method for everything in materials science. Bull. Chem. Soc. Jpn. *97*, uoad001. https://doi.org/10.1093/bulcsj/uoad001.
7. Jia, Y., and Li, J. (2019). Reconstitution of $F_oF_1$-ATPase-based biomimetic systems. Nat. Rev. Chem *3*, 361–374. https://doi.org/10.1038/s41570-019-0100-8.
8. Ariga, K., Jia, X., Song, J., Hill, J.P., Leong, D.T., Jia, Y., and Li, J. (2020). Nanoarchitectonics beyond self-assembly: challenges to create bio-like hierarchic organization. Angew. Chem. Int. Ed. *59*, 15424–15446. https://doi.org/10.1002/anie.202000802.
9. Aono, M., and Ariga, K. (2016). The way to nanoarchitectonics and the way of nanoarchitectonics. Adv. Mater. *28*, 989–992. https://doi.org/10.1002/adma.201502868.
10. Ariga, K., Li, J., Fei, J., Ji, Q., and Hill, J.P. (2016). Nanoarchitectonics for dynamic functional materials from atomic-/molecular-level manipulation to macroscopic action. Adv. Mater. *28*, 1251–1286. https://doi.org/10.1002/adma.201502545.
11. Ishii, M., Yamashita, Y., Watanabe, S., Ariga, K., and Takeya, J. (2023). Doping of molecular semiconductors through proton-coupled electron transfer. Nature *622*, 285–291. https://doi.org/10.1038/s41586-023-06504-8.
12. Stock, D., Leslie, A.G., and Walker, J.E. (1999). Molecular architecture of the rotary motor in ATP synthase. Science *286*, 1700–1705. https://doi.org/10.1126/science.286.5445.1700.
13. Mitchell, P. (1961). Coupling of phosphorylation to electron and hydrogen transfer by a chemi-osmotic type of mechanism. Nature *191*, 144–148.

14. Xu, Y., Yu, F., Jia, Y., Xu, X., and Li, J. (2024). Artificial mitochondria nanoarchitectonics via a supramolecular assembled microreactor covered by ATP synthase. Angew. Chem. Int. Ed. *63*, e202411164. https://doi.org/10.1002/anie.202411164.

15. Wang, T., Fei, J., Yu, F., Xu, X., Cui, Y., and Li, J. (2024). Nanoarchitectonics of vesicle microreactors for oscillating ATP synthesis and hydrolysis. Angew. Chem. Int. Ed. *63*, e202411981. https://doi.org/10.1002/anie.202411981.

16. Wang, T., Fei, J., Dong, Z., Yu, F., and Li, J. (2024). Nanoarchitectonics with a membrane-embedded electron shuttle mimics the bioenergy anabolism of mitochondria. Angew. Chem. Int. Ed. *63*, e202319116. https://doi.org/10.1002/anie.202319116.

17. Hahn, A., Vonck, J., Mills, D.J., Meier, T., and Kühlbrandt, W. (2018). Structure, mechanism, and regulation of the chloroplast ATP synthase. Science *360*, eaat4318. https://doi.org/10.1126/science.aat4318.

18. Duan, L., He, Q., Wang, K., Yan, X., Cui, Y., Möhwald, H., and Li, J. (2007). Adenosine triphosphate biosynthesis catalyzed by $F_oF_1$ ATP synthase assembled in polymer microcapsules. Angew. Chem. Int. Ed. *46*, 6996–7000. https://doi.org/10.1002/anie.200700331.

19. Li, Z., Yu, F., Xu, X., Wang, T., Fei, J., Hao, J., and Li, J. (2023). Photozyme-catalyzed ATP generation based on ATP synthase-reconstituted nanoarchitectonics. J. Am. Chem. Soc. *145*, 20907–20912. https://doi.org/10.1021/jacs.3c06090.

20. Martin, W., and Russell, M.J. (2007). On the origin of biochemistry at an alkaline hydrothermal vent. Philos. Trans. R. Soc. Lond. B Biol. Sci. *362*, 1887–1925. https://doi.org/10.1098/rstb.2006.1881.

21. Mahendrarajah, T.A., Moody, E.R.R., Schrempf, D., Szánthó, L.L., Dombrowski, N., Davín, A.A., Pisani, D., Donoghue, P.C.J., Szöllősi, G.J., Williams, T.A., and Spang, A. (2023). ATP synthase evolution on a cross-braced dated tree of life. Nat. Commun. *14*, 7456. https://doi.org/10.1038/s41467-023-42924-w.

22. Moody, E.R.R., Álvarez-Carretero, S., Mahendrarajah, T.A., Clark, J.W., Betts, H.C., Dombrowski, N., Szánthó, L.L., Boyle, R.A., Daines, S., Chen, X., et al. (2024). The nature of the last universal common ancestor and its impact on the early Earth system. Nat. Ecol. Evol. *8*, 1654–1666. https://doi.org/10.1038/s41559-024-02461-1.

23. Williams, T.A., Foster, P.G., Cox, C.J., and Embley, T.M. (2013). An archaeal origin of eukaryotes supports only two primary domains of life. Nature *504*, 231–236. https://doi.org/10.1038/nature12779.

24. Coleman, G.A., Davín, A.A., Mahendrarajah, T.A., Szánthó, L.L., Spang, A., Hugenholtz, P., Szöllősi, G.J., and Williams, T.A. (2021). A rooted phylogeny resolves early bacterial evolution. Science *372*, eabe0511. https://doi.org/10.1126/science.abe0511.

25. Koga, Y., Kyuragi, T., Nishihara, M., and Sone, N. (1998). Did archaeal and bacterial cells arise independently from noncellular precursors? A hypothesis stating that the advent of membrane phospholipid with enantiomeric glycerophosphate backbones caused the separation of the two lines of descent. J. Mol. Evol. *46*, 54–63. https://doi.org/10.1007/PL00006283.

26. Villanueva, L., Damsté, J.S.S., and Schouten, S. (2014). A re-evaluation of the archaeal membrane lipid biosynthetic pathway. Nat. Rev. Microbiol. *12*, 438–448. https://doi.org/10.1038/nrmicro3260.

27. Zhang, Y.-M., and Rock, C.O. (2008). Membrane lipid homeostasis in bacteria. Nat. Rev. Microbiol. *6*, 222–233. https://doi.org/10.1038/nrmicro1839.

28. Lloyd, C.T., Iwig, D.F., Wang, B., Cossu, M., Metcalf, W.W., Boal, A.K., and Booker, S.J. (2022). Discovery, structure and mechanism of a tetraether lipid synthase. Nature *609*, 197–203. https://doi.org/10.1038/s41586-022-05120-2.

29. Albers, S.-V., and Meyer, B.H. (2011). The archaeal cell envelope. Nat. Rev. Microbiol. *9*, 414–426. https://doi.org/10.1038/nrmicro2576.

30. Geisberger, T., Diederich, P., Kaiser, C.J.O., Vogele, K., Ruf, A., Seitz, C., Simmel, F., Eisenreich, W., Schmitt-Kopplin, P., and Huber, C. (2023). Formation of vesicular structures from fatty acids formed under simulated volcanic hydrothermal conditions. Sci. Rep. *13*, 15227. https://doi.org/10.1038/s41598-023-42552-w.

31. Jordan, S.F., Rammu, H., Zheludev, I.N., Hartley, A.M., Maréchal, A., and Lane, N. (2019). Promotion of protocell self-assembly from mixed amphiphiles at the origin of life. Nat. Ecol. Evol. *3*, 1705–1714. https://doi.org/10.1038/s41559-019-1015-y.

32. Szostak, J.W., Bartel, D.P., and Luisi, P.L. (2001). Synthesizing life. Nature *409*, 387–390. https://doi.org/10.1038/35053176.

33. Mansy, S.S., Schrum, J.P., Krishnamurthy, M., Tobé, S., Treco, D.A., and Szostak, J.W. (2008). Template-directed synthesis of a genetic polymer in a model protocell. Nature *454*, 122–125. https://doi.org/10.1038/nature07018.

34. Adamala, K., and Szostak, J.W. (2013). Nonenzymatic template-directed RNA synthesis inside model protocells. Science *342*, 1098–1100. https://doi.org/10.1126/science.1241888.

35. Pulletikurti, S., Veena, K.S., Yadav, M., Deniz, A.A., and Krishnamurthy, R. (2024). Experimentally modeling the emergence of prebiotically plausible phospholipid vesicles. Chem *10*, 1839–1867. https://doi.org/10.1016/j.chempr.2024.02.007.

36. Hoshino, Y., and Villanueva, L. (2023). Four billion years of microbial terpenome evolution. FEMS Microbiol. Rev. *47*, fuad008. https://doi.org/10.1093/femsre/fuad008.

37. Bonfio, C., Godino, E., Corsini, M., Fabrizi de Biani, F., Guella, G., and Mansy, S.S. (2018). Prebiotic iron–sulfur peptide catalysts generate a pH gradient across model membranes of late protocells. Nat. Catal. *1*, 616–623. https://doi.org/10.1038/s41929-018-0116-3.

38. Liu, L., Zou, Y., Bhattacharya, A., Zhang, D., Lang, S.Q., Houk, K.N., and Devaraj, N.K. (2020). Enzyme-free synthesis of natural phospholipids in water. Nat. Chem. *12*, 1029–1034. https://doi.org/10.1038/s41557-020-00559-0.

39. Chen, I.A., and Szostak, J.W. (2004). Membrane growth can generate a transmembrane pH gradient in fatty acid vesicles. Proc. Natl. Acad. Sci. USA *101*, 7965–7970. https://doi.org/10.1073/pnas.0308045101.

40. Martin, W., Baross, J., Kelley, D., and Russell, M.J. (2008). Hydrothermal vents and the origin of life. Nat. Rev. Microbiol. *6*, 805–814. https://doi.org/10.1038/nrmicro1991.

41. Kelley, D.S., Karson, J.A., Blackman, D.K., Früh-Green, G.L., Butterfield, D.A., Lilley, M.D., Olson, E.J., Schrenk, M.O., Roe, K.K., Lebon, G.T., et al. (2001). An off-axis hydrothermal vent field near the mid-atlantic ridge at 30° N. Nature *412*, 145–149. https://doi.org/10.1038/35084000.

42. Robert, F., and Chaussidon, M. (2006). A palaeotemperature curve for the Precambrian oceans based on silicon isotopes in cherts. Nature *443*, 969–972. https://doi.org/10.1038/nature05239.

43. Gaucher, E.A., Govindarajan, S., and Ganesh, O.K. (2008). Palaeotemperature trend for Precambrian life inferred from resurrected proteins. Nature *451*, 704–707. https://doi.org/10.1038/nature06510.

44. Galili, N., Shemesh, A., Yam, R., Brailovsky, I., Sela-Adler, M., Schuster, E.M., Collom, C., Bekker, A., Planavsky, N., Macdonald, F.A., et al. (2019). The geologic history of seawater oxygen isotopes from marine iron oxides. Science *365*, 469–473. https://doi.org/10.1126/science.aaw9247.

45. Kelley, D.S., Karson, J.A., Früh-Green, G.L., Yoerger, D.R., Shank, T.M., Butterfield, D.A., Hayes, J.M., Schrenk, M.O., Olson, E.J., Proskurowski, G., et al. (2005). A serpentinite-hosted ecosystem: the lost city hydrothermal field. Science *307*, 1428–1434. https://doi.org/10.1126/science.1102556.

46. Halevy, I., and Bachan, A. (2017). The geologic history of seawater pH. Science *355*, 1069–1071. https://doi.org/10.1126/science.aal4151.

47. Lang, S.Q., and Brazelton, W.J. (2020). Habitability of the marine serpentinite subsurface: a case study of the Lost City hydrothermal field. Phil. Trans. Roy. Soc. Lond. A *378*, 20180429. https://doi.org/10.1098/rsta.2018.0429.

48. Colman, D.R., Kraus, E.A., Thieringer, P.H., Rempfert, K., Templeton, A.S., Spear, J.R., and Boyd, E.S. (2022). Deep-branching acetogens in serpentinized subsurface fluids of Oman. Proc. Natl. Acad. Sci. USA *119*, e2206845119. https://doi.org/10.1073/pnas.2206845119.
49. Sleep, N.H., Bird, D.K., and Pope, E.C. (2011). Serpentinite and the dawn of life. Philos. Trans. R. Soc. Lond. B Biol. Sci. *366*, 2857–2869. https://doi.org/10.1098/rstb.2011.0129.
50. Matreux, T., Aikkila, P., Scheu, B., Braun, D., and Mast, C.B. (2024). Heat flows enrich prebiotic building blocks and enhance their reactivity. Nature *628*, 110–116. https://doi.org/10.1038/s41586-024-07193-7.
51. Yoshida, M., Muneyuki, E., and Hisabori, T. (2001). ATP synthase — a marvellous rotary engine of the cell. Nat. Rev. Mol. Cell Biol. *2*, 669–677. https://doi.org/10.1038/35089509.
52. Wang, H., and Oster, G. (1998). Energy transduction in the $F_1$ motor of ATP synthase. Nature *396*, 279–282. https://doi.org/10.1038/24409.
53. He, D., Wang, X., Yang, Y., He, R., Zhong, H., Wang, Y., Han, B., and Jin, F. (2021). Hydrothermal synthesis of long-chain hydrocarbons up to $C_{24}$ with $NaHCO_3$-assisted stabilizing cobalt. Proc. Natl. Acad. Sci. USA *118*, e2115059118. https://doi.org/10.1073/pnas.2115059118.
54. Podolsky, K.A., and Devaraj, N.K. (2021). Synthesis of lipid membranes for artificial cells. Nat. Rev. Chem *5*, 676–694. https://doi.org/10.1038/s41570-021-00303-3.
55. Purvis, G., Šiller, L., Crosskey, A., Vincent, J., Wills, C., Sheriff, J., Xavier, C., and Telling, J. (2024). Generation of long-chain fatty acids by hydrogen-driven bicarbonate reduction in ancient alkaline hydrothermal vents. Commun. Earth Environ. *5*, 30. https://doi.org/10.1038/s43247-023-01196-4.
56. Beyazay, T., Ochoa-Hernández, C., Song, Y., Belthle, K.S., Martin, W.F., and Tüysüz, H. (2023). Influence of composition of nickel-iron nanoparticles for abiotic CO2 conversion to early prebiotic organics. Angew. Chem. Int. Ed. *62*, e202218189. https://doi.org/10.1002/anie.202218189.
57. Zhu, P., Wang, C., Lang, J., He, D., and Jin, F. (2024). Prebiotic synthesis of microdroplets from formate over a bimetallic cobalt–nickel nanomotif. J. Am. Chem. Soc. *146*, 25005–25015. https://doi.org/10.1021/jacs.4c06989.
58. Deamer, D. (2017). The role of lipid membranes in life's origin. Life *7*, 5.
59. Zilberstein, D., Agmon, V., Schuldiner, S., and Padan, E. (1984). Escherichia coli intracellular pH, membrane potential, and cell growth. J. Bacteriol. *158*, 246–252. https://doi.org/10.1128/jb.158.1.246-252.1984.
60. Tran, Q.H., and Unden, G. (1998). Changes in the proton potential and the cellular energetics of Escherichia coli during growth by aerobic and anaerobic respiration or by fermentation. Eur. J. Biochem. *251*, 538–543. https://doi.org/10.1046/j.1432-1327.1998.2510538.x.
61. Bartelds, R., Nematollahi, M.H., Pols, T., Stuart, M.C.A., Pardakhty, A., Asadikaram, G., and Poolman, B. (2018). Niosomes, an alternative for liposomal delivery. PLoS One *13*, e0194179.
62. Clement, N.R., and Gould, J.M. (1981). Pyranine (8-hydroxy-1,3,6-pyrenetrisulfonate) as a probe of internal aqueous hydrogen ion concentration in phospholipid vesicles. Biochemistry *20*, 1534–1538. https://doi.org/10.1021/bi00509a019.
63. Kuyper, C.L., Kuo, J.S., Mutch, S.A., and Chiu, D.T. (2006). Proton permeation into single vesicles occurs via a sequential two-step mechanism and is heterogeneous. J. Am. Chem. Soc. *128*, 3233–3240. https://doi.org/10.1021/ja057349c.
64. Paradies, G., Paradies, V., De Benedictis, V., Ruggiero, F.M., and Petrosillo, G. (2014). Functional role of cardiolipin in mitochondrial bioenergetics. Biochim. Biophys. Acta *1837*, 408–417. https://doi.org/10.1016/j.bbabio.2013.10.006.
65. Apel, C.L., Deamer, D.W., and Mautner, M.N. (2002). Self-assembled vesicles of monocarboxylic acids and alcohols: conditions for stability and for the encapsulation of biopolymers. Biochim. Biophys. Acta *1559*, 1–9. https://doi.org/10.1016/S0005-2736(01)00400-X.
66. Hargreaves, W.R., and Deamer, D.W. (1978). Liposomes from ionic, single-chain amphiphiles. Biochemistry *17*, 3759–3768. https://doi.org/10.1021/bi00611a014.
67. Baross, J.A., and Hoffman, S.E. (1985). Submarine hydrothermal vents and associated gradient environments as sites for the origin and evolution of life. Orig. Life Evol. Biosph. *15*, 327–345. https://doi.org/10.1007/BF01808177.
68. Schwander, L., Brabender, M., Mrnjavac, N., Wimmer, J.L.E., Preiner, M., and Martin, W.F. (2023). Serpentinization as the source of energy, electrons, organics, catalysts, nutrients and pH gradients for the origin of LUCA and life. Front. Microbiol. *14*, 1257597. https://doi.org/10.3389/fmicb.2023.1257597.
69. Levental, I., and Lyman, E. (2023). Regulation of membrane protein structure and function by their lipid nano-environment. Nat. Rev. Mol. Cell Biol. *24*, 107–122. https://doi.org/10.1038/s41580-022-00524-4.
70. He, W., Song, H., Su, Y., Geng, L., Ackerson, B.J., Peng, H.B., and Tong, P. (2016). Dynamic heterogeneity and non-Gaussian statistics for acetylcholine receptors on live cell membrane. Nat. Commun. *7*, 11701. https://doi.org/10.1038/ncomms11701.
71. Pasupuleti, M., Schmidtchen, A., and Malmsten, M. (2012). Antimicrobial peptides: key components of the innate immune system. Crit. Rev. Biotechnol. *32*, 143–171. https://doi.org/10.3109/07388551.2011.594423.
72. Jeong, J.-H., Han, J.S., Jung, Y., Lee, S.M., Park, S.H., Park, M., Shin, M.G., Kim, N., Kang, M.S., Kim, S., et al. (2023). A new AMPK isoform mediates glucose-restriction induced longevity non-cell autonomously by promoting membrane fluidity. Nat. Commun. *14*, 288. https://doi.org/10.1038/s41467-023-35952-z.
73. Budin, I., Debnath, A., and Szostak, J.W. (2012). Concentration-Driven Growth of Model Protocell Membranes. J. Am. Chem. Soc. *134*, 20812–20819. https://doi.org/10.1021/ja310382d.
74. Mahapatra, A., Mandal, N., and Chattopadhyay, K. (2021). Cholesterol in Synaptic Vesicle Membranes Regulates the Vesicle-Binding, Function, and Aggregation of α-Synuclein. J. Phys. Chem. B *125*, 11099–11111. https://doi.org/10.1021/acs.jpcb.1c03533.
75. Walker, J.E. (2013). The ATP synthase: the understood, the uncertain and the unknown. Biochem. Soc. Trans. *41*, 1–16. https://doi.org/10.1042/BST20110773.
76. Orgel, L.E. (1999). Are you serious, Dr Mitchell? Nature *402*, 17. https://doi.org/10.1038/46903.
77. Lane, N., Allen, J.F., and Martin, W. (2010). How did LUCA make a living? Chemiosmosis in the origin of life. Bioessays *32*, 271–280. https://doi.org/10.1002/bies.200900131.
78. Bowman, J.C., Petrov, A.S., Frenkel-Pinter, M., Penev, P.I., and Williams, L.D. (2020). Root of the tree: the significance, evolution, and origins of the ribosome. Chem. Rev. *120*, 4848–4878. https://doi.org/10.1021/acs.chemrev.9b00742.
79. Muchowska, K.B., Varma, S.J., and Moran, J. (2019). Synthesis and breakdown of universal metabolic precursors promoted by iron. Nature *569*, 104–107. https://doi.org/10.1038/s41586-019-1151-1.
80. Brabender, M., Henriques Pereira, D.P., Mrnjavac, N., Schlikker, M.L., Kimura, Z.I., Sucharitakul, J., Kleinermanns, K., Tüysüz, H., Buckel, W., Preiner, M., and Martin, W.F. (2024). Ferredoxin reduction by hydrogen with iron functions as an evolutionary precursor of flavin-based electron bifurcation. Proc. Natl. Acad. Sci. USA *121*, e2318969121. https://doi.org/10.1073/pnas.2318969121.
81. Preiner, M., Igarashi, K., Muchowska, K.B., Yu, M., Varma, S.J., Kleinermanns, K., Nobu, M.K., Kamagata, Y., Tüysüz, H., Moran, J., and Martin, W.F. (2020). A hydrogen-dependent geochemical analogue of primordial carbon and energy metabolism. Nat. Ecol. Evol. *4*, 534–542. https://doi.org/10.1038/s41559-020-1125-6.

Cell Reports Physical Science, Volume *6*

## Supplemental information

# Chemiosmotic ATP synthesis by minimal protocells

**Fanchen Yu, Jinbo Fei, Yi Jia, Tonghui Wang, William F. Martin, and Junbai Li**

**Table of Contents**

Supplemental Methods
- Materials
- Vesicles
- pH gradients
- Turbidity
- Membrane fluidity
- Purification of ATP synthase
- Reconstitution of ATP synthase
- Synthesis of ATP
- Characterization

Supplemental Items
- Figure S1. Encapsulation of pH fluorescent probes in vesicles and formation of pH gradients.
- Figure S2. Determination of solution pH through the pH fluorescent probe.
- Figure S3. The $C_{18:1}$ vesicles cannot maintain proton gradient.
- Figure S4. Dynamic light scattering of various vesicles before and after acid bath.
- Figure S5. Resistance of fatty acid vesicles to pH changes.
- Figure S6. The resistance of $C_{18}$ or $C_{18}$-$C_{18}$OH vesicles to acid bath.
- Figure S7. Excitation spectra of HPTS inside vesicles after acid bath over time.
- Figure S8. The resistance of other fatty acid vesicles to acid bath.
- Figure S9. The relationship between the formation of vesicles and temperature.
- Figure S10. The influence of temperature and concentration on vesicles
- Figure S11. The GP value of vesicles assembled with fatty acids/alcohols or phospholipids.
- Figure S12. The influence of temperature on membrane fluidity.
- Figure S13. Verification of structure and function of ATP synthase.

Supplemental References

## Supplemental Methods

### Materials

1,2-dimyristoyl-sn-glycero-3-phosphocholine ($dC_{14}$, DMPC) was purchased from Avanti. 1,2-dipalmitoyl-sn-glycero-3-phosphocholine ($dC_{16}$, DPPC) and 1,2-distearoyl-sn-glycero-3-phosphocholine ($dC_{18}$, DSPC) were purchased from Sigma-Aldrich. 1,2-dioleoyl-sn-glycero-3-phosphocholine ($dC_{18:1}$, DOPC), 1′,3′-bis[1,2-dioleoyl-sn-glycero-3-phospho]-glycerol sodium salt ($qC_{18:1}$, cardiolipin), oleic acid ($C_{18:1}$), tetradecanol ($C_{14}OH$), monomyristin ($C_{14}G$) and phytol ($C_{20:br}OH$) was purchased from Macklin. Stearic acid ($C_{18}$) was purchased from Aladdin (Shanghai) Reagent Co., LTD. Phytanic acid ($C_{20:br}$) was purchased from Kaiwei Chemical. HPTS was purchased from Acros Organics. Laurdan was purchased from Macklin. 6-tetramethylrhodamine isothiocyanate (TRITC) was purchased from Avanti. Sephadex G-50 was purchased from JandK Scientific. Di(adenosine-5')pentaphosphate trilithium salt ($Ap_5A$) was purchased from Shanghai yuanye Bio-Technology Co., Ltd. Oligomycin was purchased from Aladdin Reagent (Shanghai) Co., LTD. Ethylenediaminetetracetic acid disodium salt ($EDTA\text{-}Na_2$), sodium dihydrogen phosphate ($NaH_2PO_4$), magnesium chloride ($MgCl_2$), manganese sulfate ($MnSO_4$) and ammonium hydrogen carbonate ($NH_4HCO_3$) were purchased from Aladdin Reagent (Shanghai) Co., LTD. Dithiothreitol (DTT) was bought from Merck. Luciferase Assay System was from Promega. Triton X-100, adenosine-5’-triphosphonic acid disodium salt (ATP $Na_2$), β-D-octyiglucoside and Adenosine-5’-diphosphate disodium salt (ADP $Na_2$) were purchased from Solarium. SDS-PAGE Gel Preparation Kit was obatined from Beyotime. Coomassie brilliant blue G-250 was from Solarbio Biotechnology Co., LTD. Bio-Beads SM-2 was purchased from BIO-RAD. All chemicals were used directly without further purification. Deionized water (18.2 MΩ·cm) was obtained by ELGA PURELAB (U.K.)

### Vesicles

All vesicles were prepared by thin-film hydration method. In detail, each membrane component was dissolved with 2 mL of chloroform in a round-bottom flask. The concentration of fatty-acid tail chain in every vesicle was set at 1 mM. Next, uniform film formed at the bottom of the flask by a rotary evaporator at 55 °C for phospholipids (above the phase transition temperature) or Tm - 10 °C for fatty acids, and was placed in a vacuum over overnight. Then, 2 mL of hydration solution (10 mM $K_2HPO_4$, 0.1 mM KOH, pH 9.5) was added to keep hydration for 2 h at 70 °C, a temperature of alkaline hydrothermal fluid.[1] The vesicles were repeatedly extruded through the PC membrane with a pore size of 200 nm in the preheated Mini-extruder to obtain uniform unilamellar vesicles for pH gradient and ATP synthesis experiments. The vesicles used for microscopy were not extruded.

### pH gradients

In the method of preparing vesicles, 2 mL of hydration solution containing pH probes (10 mM $K_2HPO_4$, 1 mM KOH, 1 mM HPTS, pH 9.5) was added to keep hydration for 2 h at 70 °C.

To obtain vesicles containing pH probes, 1 mL of vesicle solution with pH probes was took and the probe outside the vesicle was removed by size exclusion chromatography (17mm×13.4mm×305mm glass column filled with Sephadex G-50 medium beads) and elution solution (10 mM $K_2HPO_4$, 0.1 mM KOH, 2.45 mM KCl, pH 9.5). Then, the fractions were collected by a 96 well plate. To determine the location of vesicles, the absorbance at 450 nm was detected by a microplate reader. Finally, these vesicles containing the pH probes were collected. To observe these vesicles by confocal fluorescent microscopy, 50 µL of vesicles and 0.5 µL of 1 mM TRITC dye were mixed before observation.

To generate pH gradient across the vesicle membrane, 0.2 mL of vesicles and 2 mL of acid bath solution (pH 6.5, 7.5 mM $KH_2PO_4$, 2.5 mM $K_2HPO_4$, 6.25 mM KCl) were mixed and stirred for 1 min. Next, the excitation spectrum of the mixture from 380 to 500 nm was measured over time (emission at 513 nm). Finally, 1 µL of Triton X-100 was added to break the vesicles, the excitation spectrum was recorded as the pH value outside the vesicle to calculate the pH gradient, $\Delta pH(t) = pH(t) - pH(\text{Triton})$.

In our case, HPTS was used as the pH probe. As proton concentration increases, the excitation intensity at 406 nm increases while that at 460 nm decreases. The pH can be calculated from the excitation intensity ratio $I_{460}/I_{406}$ according to the standard curve, as the following equation:

$$pH = a + bx + cx^2 + dx^3 + ex^4 + fx^5$$

where x is $I_{460}/I_{406}$. The constants are a: 6.13, b: 3.20, c: -3.26, d: 2.05, e: -0.64, f: 0.079.

### Turbidity

**Turbidity titration.** Firstly, fatty acids (final concentration 100 mM) were dissolved in 0.1 M NaOH and a few microliters of 2 M HCl were added each time to decrease its pH while shaking it for 5 min. Then, measure its pH value by pH meter and its absorbance at 480 nm by UV-Vis spectrophotometer. Repeat this process until its pH<4.

**Acid bath of preformed vesicles.** Fatty acids (final concentration 100 mM) were dissolved in 0.15 M KOH to form micelles (pH>12). To form vesicles, 25 μL micelles and 475 μL pH 8.3 buffer (pH 8.3, 100 mM Bicine, 10 mM $KH_2PO_4$) were mixed in an orbital shaker (25 °C, 90 rpm). Next, mix 0.5 mL vesicles and 1.5 mL pH 6.5 buffer (pH 6.5, 100 mM Bis-Tris, 10 mM $KH_2PO_4$). Finally, the mixture's absorbance at 480 nm was measured over time.

**Acid bath of micelles.** Fatty acids (final concentration 100 mM) were dissolved in 0.15 M KOH to form micelles (pH>12). Directly mixing 25 μL micelles, 475 μL pH 8.3 buffer (pH 8.3, 100 mM Bicine, 10 mM $KH_2PO_4$) and 1.5 mL pH 6.5 buffer (pH 6.5, 100 mM Bis-Tris, 10 mM $KH_2PO_4$). Finally, the mixture's absorbance at 480 nm was measured over time.

To observe these vesicles by confocal microscopy, 50 μL vesicles and 0.5 μL 1 mM TRITC dye were mixed before observation.

### Membrane fluidity

Vesicles were prepared by thin-film hydration method. The membrane components were dissolved by 2 mL chloroform containing 5 μM membrane fluidity probes (Laurdan) in a round-bottom flask. The concentration of fatty-acid tail chain in every vesicle was set at 1 mM. Next, uniform films were obtained at the bottom of the flask by a rotary evaporator at 55 °C for phospholipids and below the melting point by 10 °C for fatty acids. They were placed in a vacuum over overnight. Through the similar procedure, unilamellar vesicles were prepared by repeatedly extruding the vesicles through the PC membrane with a pore size of 200 nm in the preheated Mini-extruder. 2 mL of hydration solution (10 mM $K_2HPO_4$, 0.1 mM KOH, 2.45 mM KCl, pH 9.5) was added and hydrated for 2 h at 70 °C. Finally, the emission spectrum of the vesicles from 400 to 600 nm was measured (excitation at 370 nm).

According to the probe's emission spectrum, GP (generalized polarization) value is calculated as the following equation:[2]

$$GP = (I_{430} - I_{500}) / (I_{430} + I_{500})$$

The higher GP value means the lower membrane fluidity.

### Purification of ATP synthase

ATP synthase was purified from chloroplasts of spinach according to the previous method.[3] Firstly, fresh spinach (500 g) was cleaned thoroughly and stored at 4 °C overnight. After adding buffer I (500 mL, pH 8.0, 2 mM $MgCl_2$, 100 mM Tricine-NaOH, 0.4 M sucrose), the spinach veined was triturated to grainy by homogenizer. This crushed mixture was filtered by cotton gauze, and the filtrate was centrifuged (10600 gmax, 30 min) to collect the precipitation, which was suspended in buffer II (pH 8.0, 0.5 mM $MgCl_2$, 10 mM Tris-HCl). After centrifuging this suspension (16900 gmax,15 min), the precipitation was dispersed in buffer III (pH 8.0, 0.5 mM $MgCl_2$, 0.4 M sucrose, 10 mM Tris-HCl) for the next centrifugation (16900 gmax for 25 min). Then after removal of the supernatant, buffer IV (pH 8.0, 0.2 mM $MgCl_2$, 0.4 M sucrose, 50 mM Tricine-NaOH) was poured into the tube to obtain a chlorophyll solution ~5 mg/mL. And the suspension was mixed with equal volume buffer V (pH 8.0, 50 mM DTT, 60 mM β-D-octylglucoside, 25 mM Na cholate, 2 mM ATP $Na_2$, 200 mM sucrose, 20 mM Tricine-NaOH, 5 mM $MgCl_2$, 400 mM $(NH_4)_2SO_4$) under stirring for 30min. After centrifugation (208000 gmax, 60 min), the suspension was injected $(NH_4)_2SO_4$ (~45%) to make the ATP synthase precipitate out, and collected by centrifugation (12000 gmax, 10 min). After mixed with buffer VI (pH 7.2, 4 mM dodecylmaltoside, 0.5 mM $Na_2EDTA$, 2 mM $MgCl_2$, 200 mM sucrose, 30 mM $NaH_2PO_4$), the crude extract was precipitated and then adding buffer VII (pH 7.2, 4 mM dodecylmaltoside, 0.5 mM $Na_2EDTA$, 30 mM $NaH_2PO_4$, 2 mM $MgCl_2$) with equal volume. After sucrose density gradient centrifugation (60%, 52%, 44%, 36%~28%, and 20%), ATP synthase was collected at 44% sucrose layer and stored in liquid nitrogen. Finally, the structure of ATP synthase was verified through SDS-PAGE (sodium dodecyl sulphate-polyacrylamide gel electrophoresis).

## Reconstitution of ATP synthase

940 μL of vesicle solution was mixed 80 μL of 10% Triton X-100 and 60 μL of ATP synthase and stirred for 1 h at room temperature. Then, Bio-Beads SM2 was added to remove Triton X-100.[3,4] The ATP synthase reconstituted vesicles were obtained.

## Synthesis of ATP

For ATP synthesis, proton gradient was generated by base bath and the ATP concentration was measured by the luciferin-luciferase assay. In detail, 100 μL of base bath solution (10 mM $K_2HPO_4$, 0.8 mM KOH, 2.45 mM KCl, 0.2 mM ADP, pH 9.5) was mixed with 100 μL of ATP synthase reconstituted vesicles (pH 7.5) and 10 μL of luciferin-luciferase. Next, photon counts of the mixture were detected by using an ultra-weak luminescence analyzer (BPCL-GP15). Finally, ATP concentration was calculated through the standard curve obtained by pure ATP solution. The detection principle of ATP in luciferin-luciferase assay is to consume ATP to emit photons:

$$\text{ATP + luciferin} \xrightarrow[\text{Mg}^{2+},\ \text{O}_2]{\text{luciferase}} \text{AMP + oxyluciferin} + \text{H}_2\text{O} + \text{CO}_2 + h\nu$$

## Characterization

Confocal laser scanning microscopy (CLSM) images were obtained by using an Olympus FV3000. The zeta potential test results were obtained from Zetasizer Nano 7S ZEN3600. ATP production was tested by Luciferin-luciferase assay using the ultra-weak luminescence analyzer (BPCL-GP15). Fluorescence excitation spectra were measured by Edinburgh FLS980 fluorescence spectrometer and Edinburgh FS5 Fluorescence Spectrometer. Absorption spectra were measured by Shimadzu UV-Vis spectrophotometer UV-3600i Plus and Thermo Scientific Muitiskan FC microplate reader.

## Supplemental Items

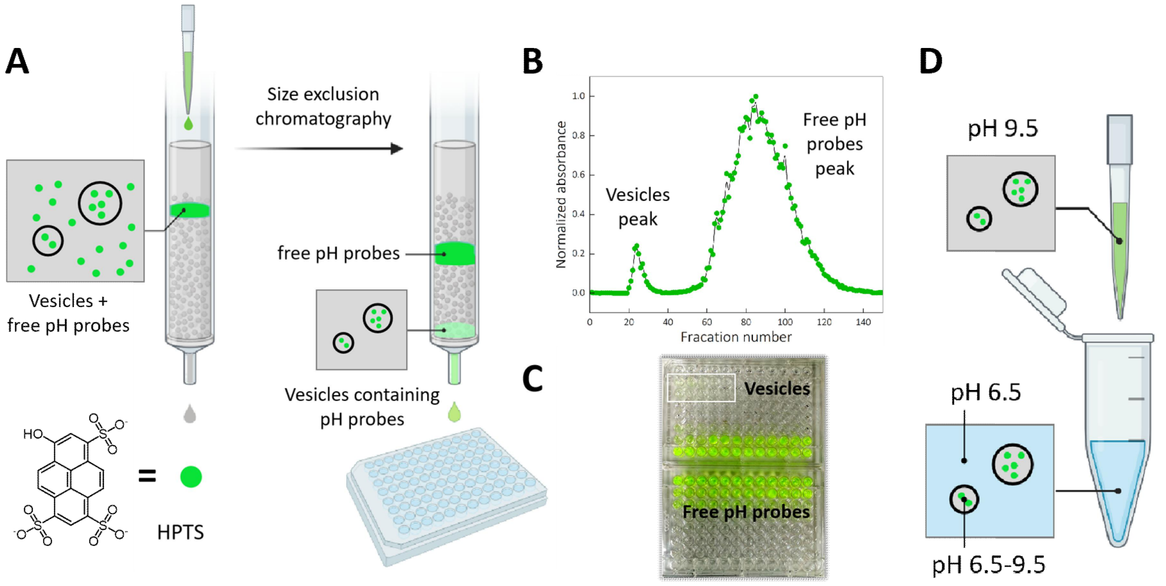


**Figure S1. Encapsulation of pH fluorescent probes in vesicles and formation of pH gradients.** (A) Schematic diagram of obtaining vesicles containing pH probes. (B) Elution curve of fractions. (C) Collecting fractions based on the vesicle peak of the elution curve. (D) Schematic diagram of forming proton gradients by acid bath.

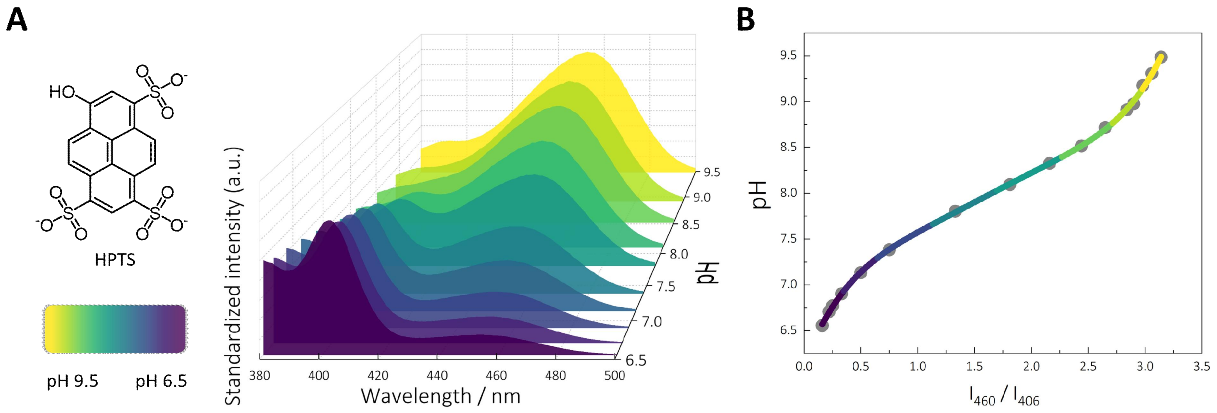


**Figure S2. Determination of solution pH through the pH fluorescent probe.** (A) Excitation spectra of HPTS over pH. (B) Standard curve of HPTS presenting pH over excitation intensity ratio **$I_{460}$/$I_{406}$.**

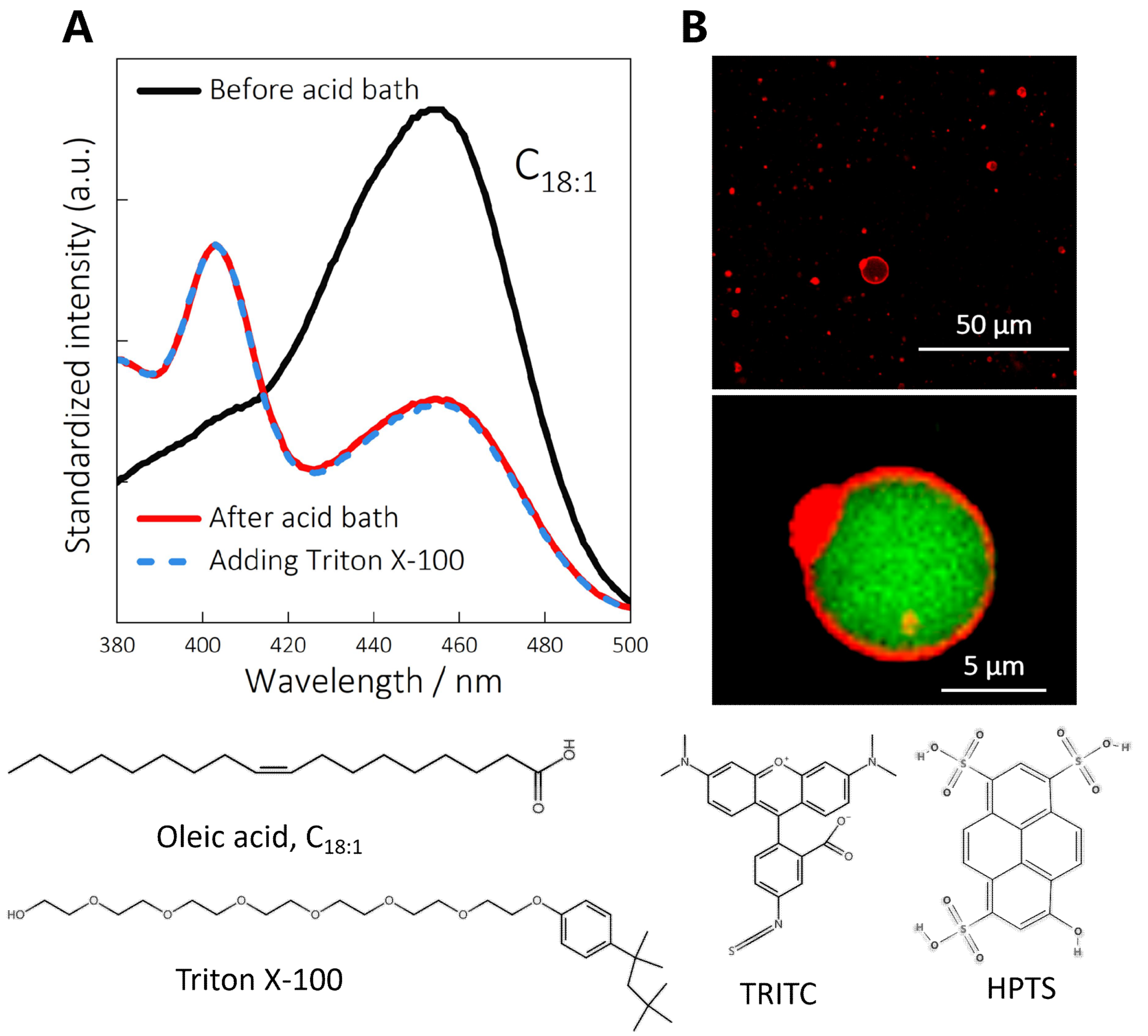


**Figure S3. The $C_{18:1}$ vesicles cannot maintain proton gradient.** (A) Fluorescence spectra of HPTS inside $C_{18:1}$ vesicles before acid bath (black line), after acid bath within 1 min (red line) and the addition of triton X-100 (bule line). (B) The lowly and highly-magnified CLSM (confocal laser scanning microscope) images of $C_{18:1}$ vesicles after acid bath for 48 h, red (TRITC), green (HPTS).

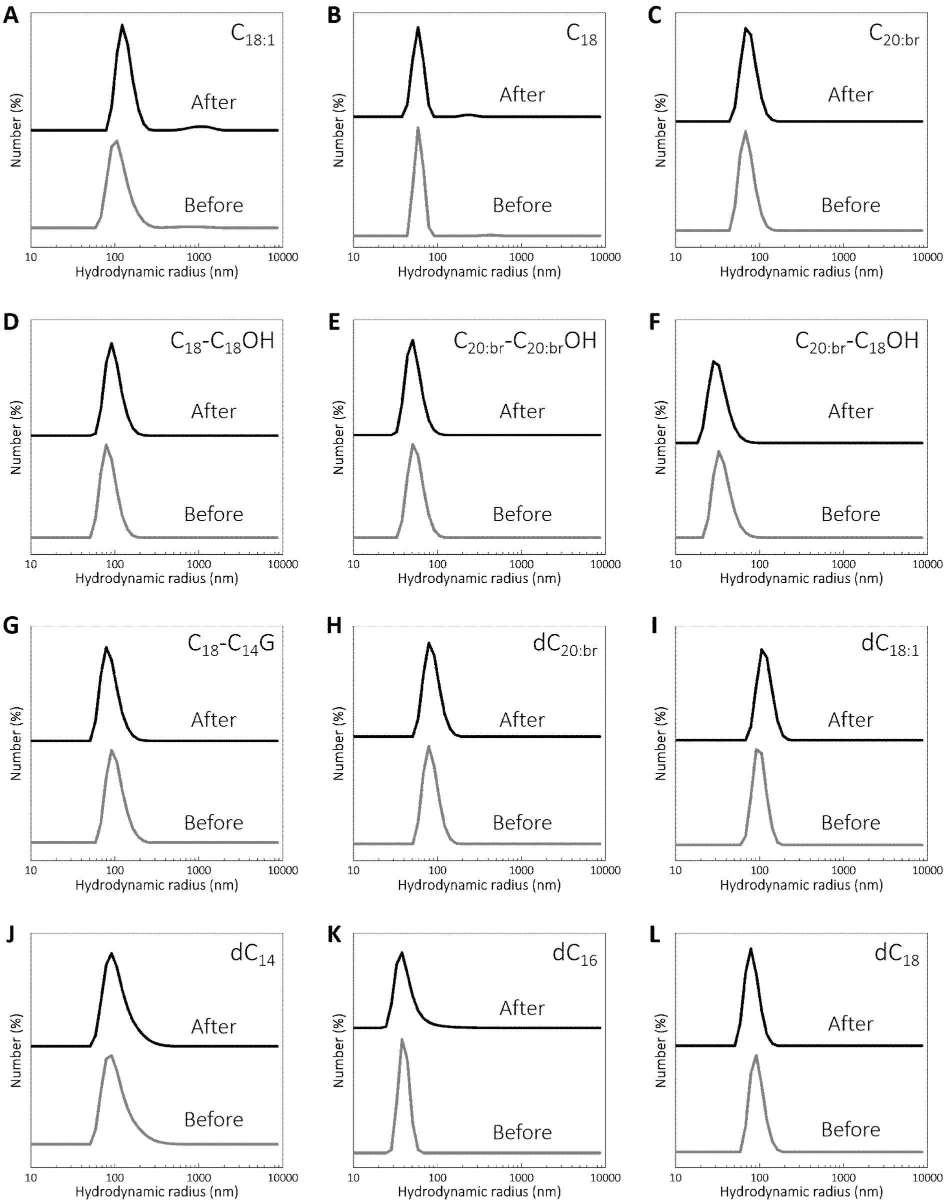


**Figure S4. Hydrodynamic radius of various vesicles before and after acid bath measured by dynamic light scattering.** The mole ratio of acid/alcohol is 2:1. (A) $C_{18:1}$; (B) $C_{18}$; (C) $C_{20:br}$; (D) $C_{18}$-$C_{18}OH$; (E) $C_{20:br}$-$C_{20:br}OH$; (F) $C_{20:br}$-$C_{18}OH$; (G) $C_{18}$-$C_{14}G$; (H) $dC_{20:br}$; (I) $dC_{18:1}$; (J) $dC_{14}$; (K) $dC_{16}$; (L) $dC_{18}$.

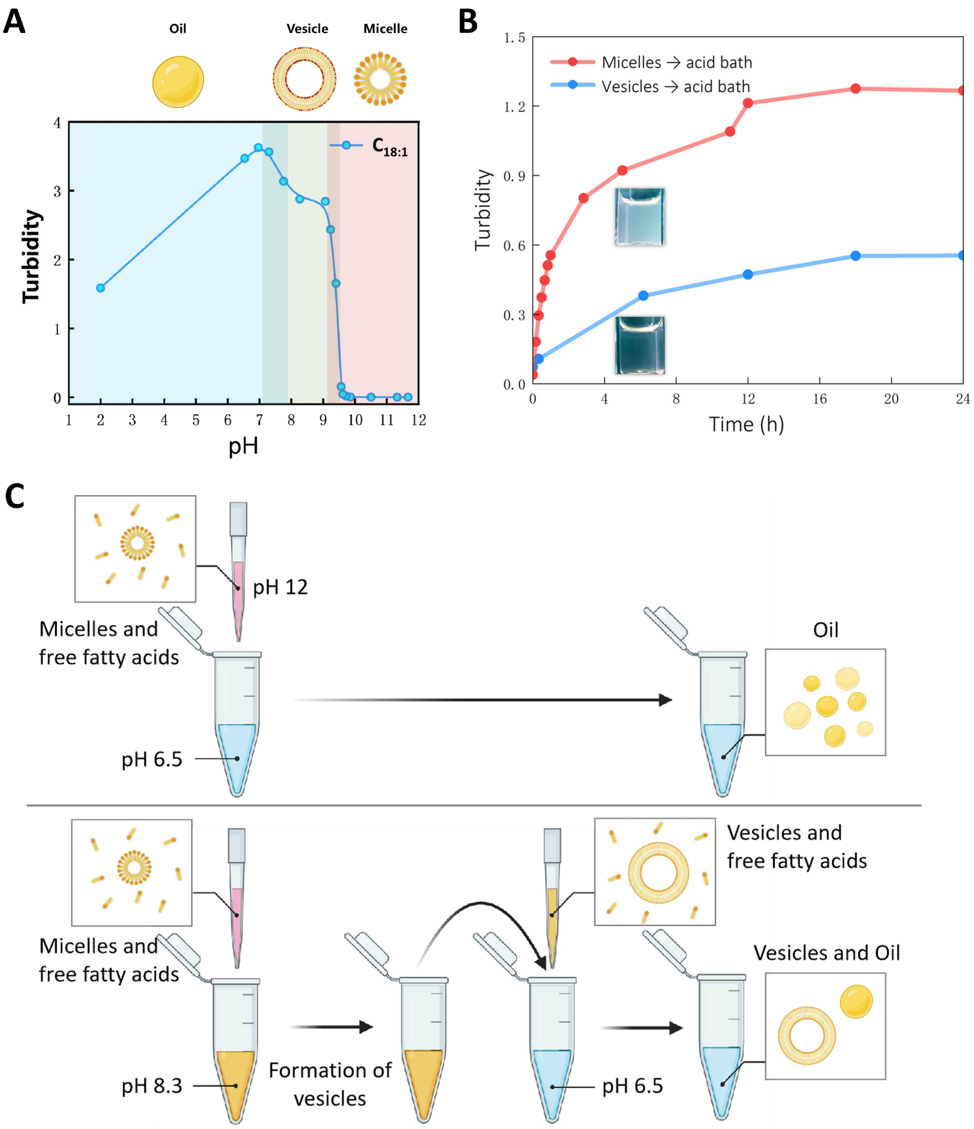


**Figure S5. Resistance of fatty acid vesicles to pH changes.** (A) Turbidity titration curve of oleic acid ($C_{18:1}$). (B) The turbidity of oleic acid micelles and preformed vesicles after acid bath (pH 6.5) over time. (C) Schematic diagram of comparison between fatty acid ($C_{18:1}$) micelles and preformed vesicles after acid bath.

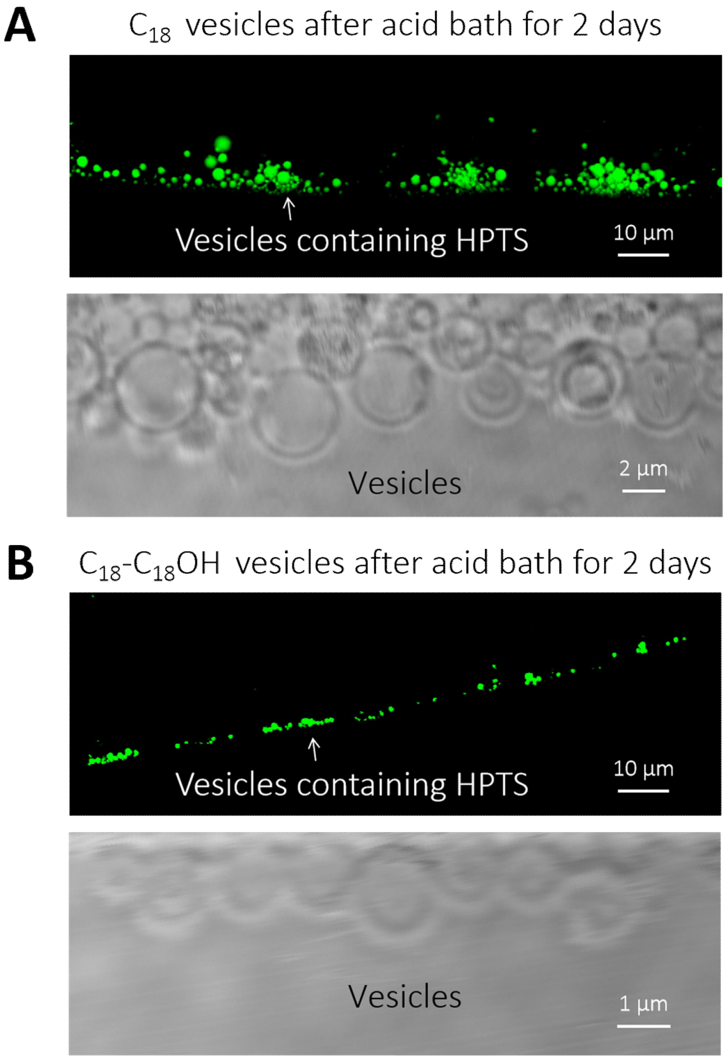


**Figure S6. The resistance of $C_{18}$ or $C_{18}$-$C_{18}OH$ vesicles to acid bath.** (A) The CLSM images of $C_{18}$ vesicles after acid bath (pH 6.5) for 2 days. Upper panel, vesicles containing HPTS (green). Lower panel, highly-magnified bright field. (B) The CLSM images of $C_{18}OH$ vesicles after acid bath for 2 days. The mole ratio of acid/alcohol is 2:1. Upper panel, vesicles containing HPTS (green). Lower panel, highly-magnified bright field.

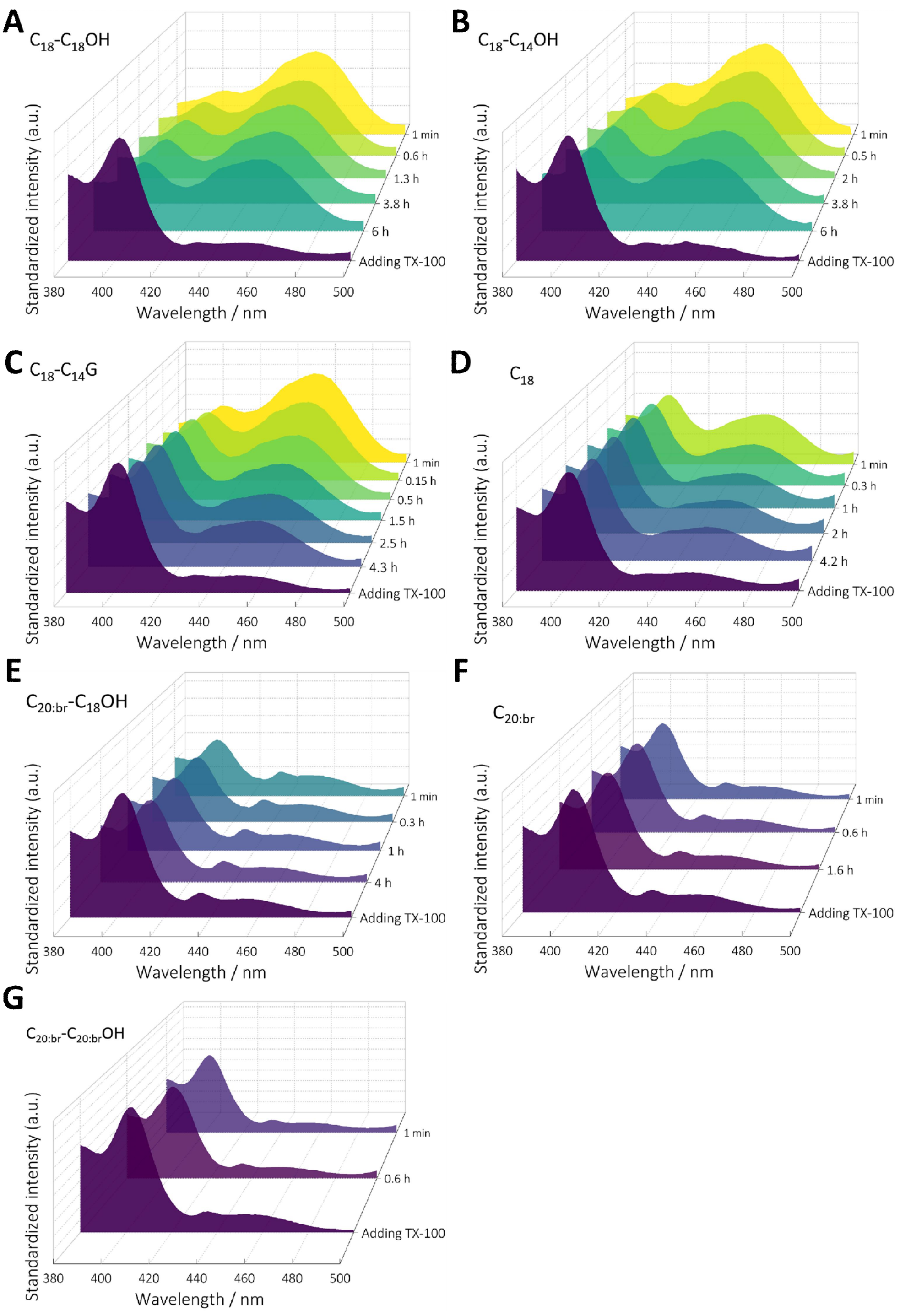


**Figure S7. Excitation spectra of HPTS inside vesicles composed of fatty acids and their derivatives after acid bath over time.** After adding triton X-100 (TX-100) to break vesicles, the pH outside the vesicles was obtained. (A) $C_{18}$-$C_{18}OH$; (B) $C_{18}$-$C_{14}OH$; (C) $C_{18}$-$C_{14}G$; (D) $C_{18}$; (E) $C_{20:br}$-$C_{18}OH$; (F) $C_{20:br}$; (G) $C_{20:br}$-$C_{20:br}OH$.

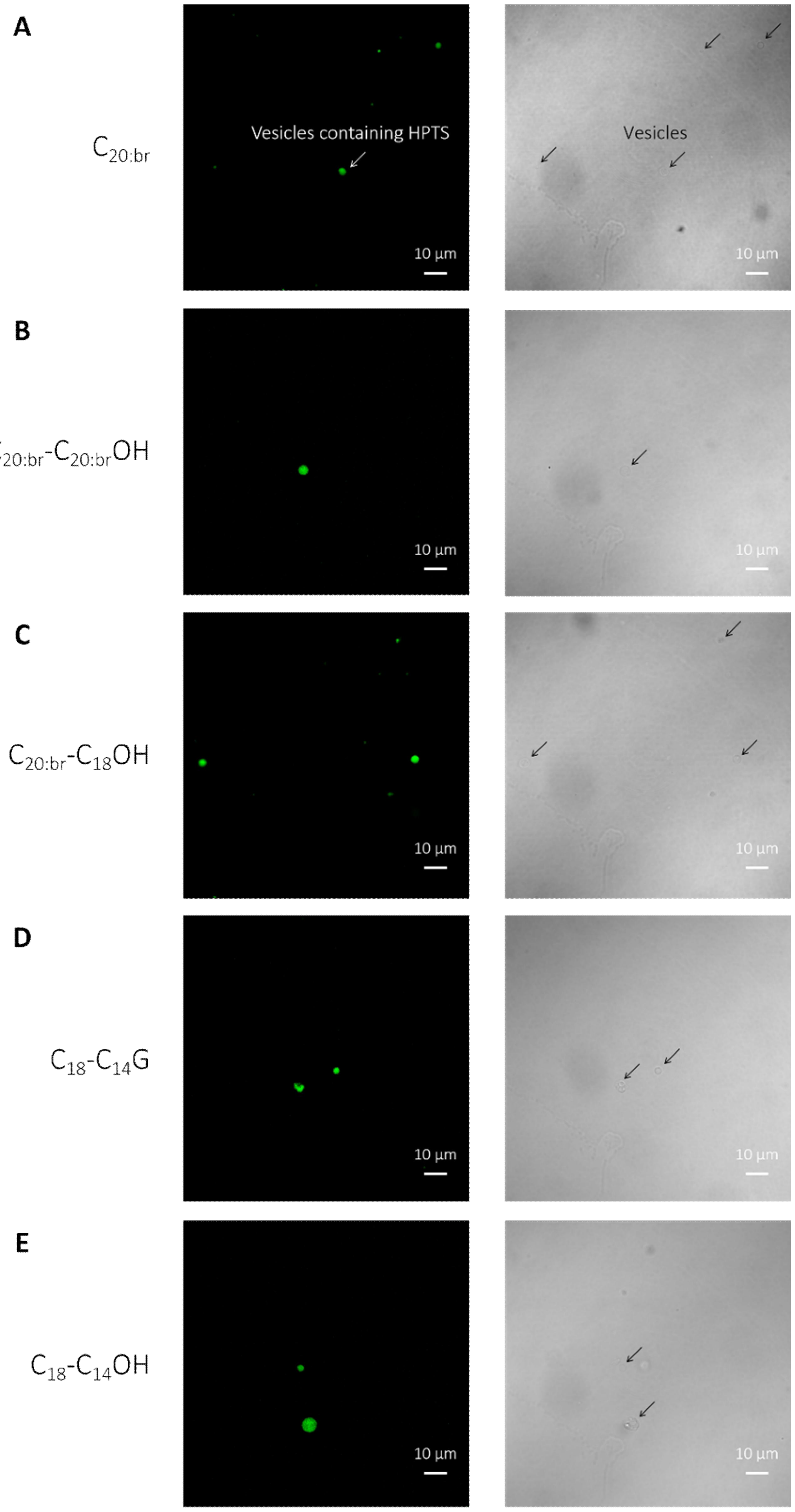


**Figure S8. The resistance of other fatty acid vesicles to acid bath.** The CLSM images of (A) $C_{20:br}$, (B) $C_{20:br}$-$C_{20:br}OH$, (C) $C_{20:br}$-$C_{18}OH$, (D) $C_{18}$-$C_{14}G$ or (E) $C_{18}$-$C_{14}OH$ vesicles after acid bath (pH 6.5) for 4 h. The mole ratio of acid/alcohol was 2:1. Left panel, vesicles containing HPTS (green). Right panel, bright field. The arrow marks the vesicles.

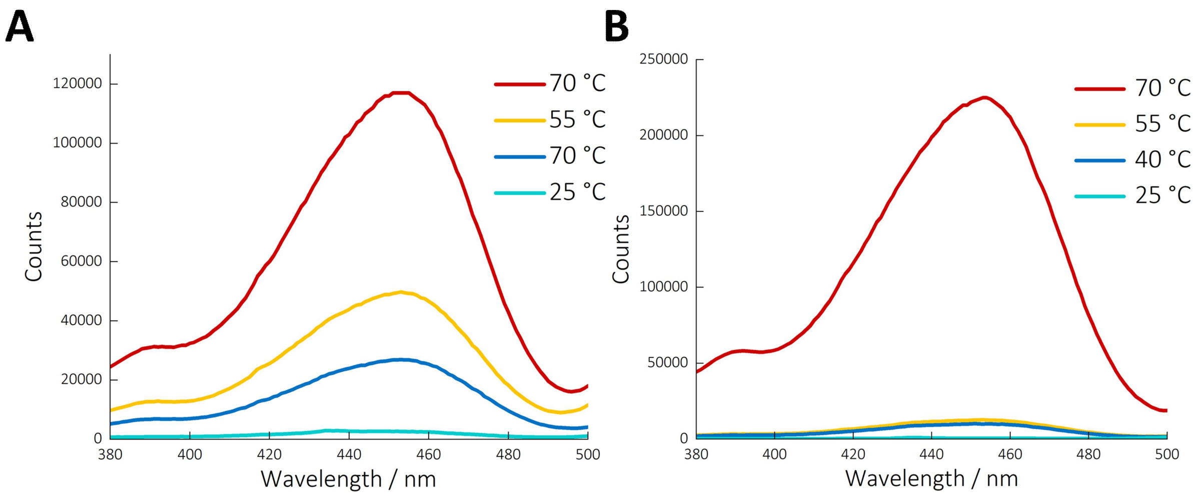


**Figure S9. The relationship between the formation of vesicles and temperature.** (A) Excitation spectra of HPTS inside $C_{18}$-$C_{18}OH$ vesicles prepared at different temperatures. (B) Excitation spectra of HPTS inside $C_{18}$ vesicles prepared at different temperatures.

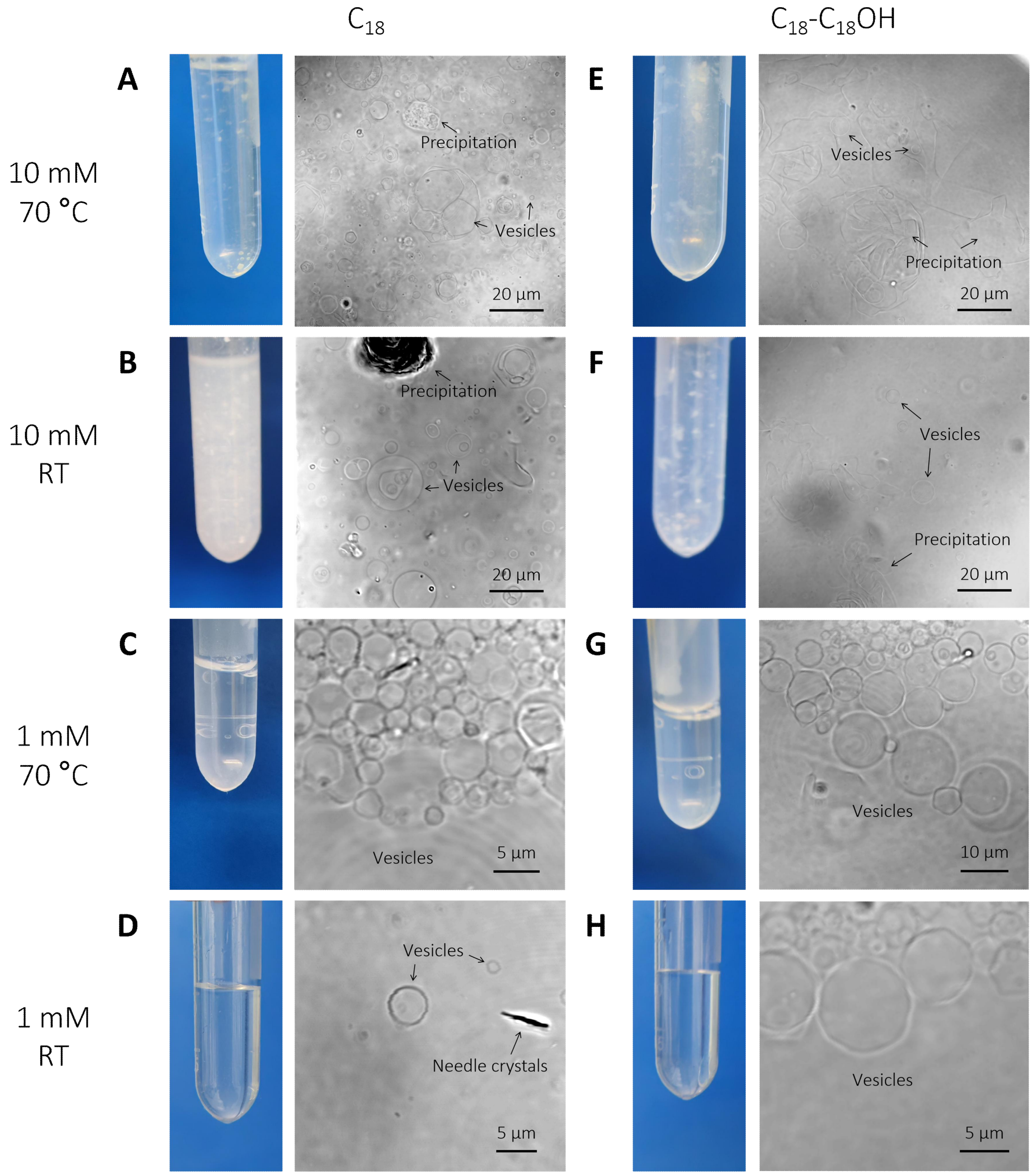


**Figure S10. The influence of temperature and concentration on vesicles.** Photos and microscopy images of $C_{18}$ (A-D) or $C_{18}$-$C_{18}OH$ (E-H) under different conditions. The molar ratio $C_{18}/C_{18}OH$ is 2:1. (A, E) When total concentration is 10 mM at 70 °C, the solution was relatively clear and transparent. Numerous vesicles and amorphous precipitation were observed by microscopy imaging. (B, F) After cooling down to room temperature (~20 °C) for 2 days, the $C_{18}$ solution became turbid and more precipitation appeared, but numerous vesicles were still present. The $C_{18}$-$C_{18}OH$ solution was relatively clear and transparent. Numerous vesicles of $C_{18}$-$C_{18}OH$ were observed. (C, D, G, H) When total concentration was 1 mM at 70 °C or after cooling down to room temperature (~20 °C) for 2 days, the solution of $C_{18}$ or $C_{18}$-$C_{18}OH$ was relatively clear and transparent. Numerous vesicles and some needle crystals were observed.

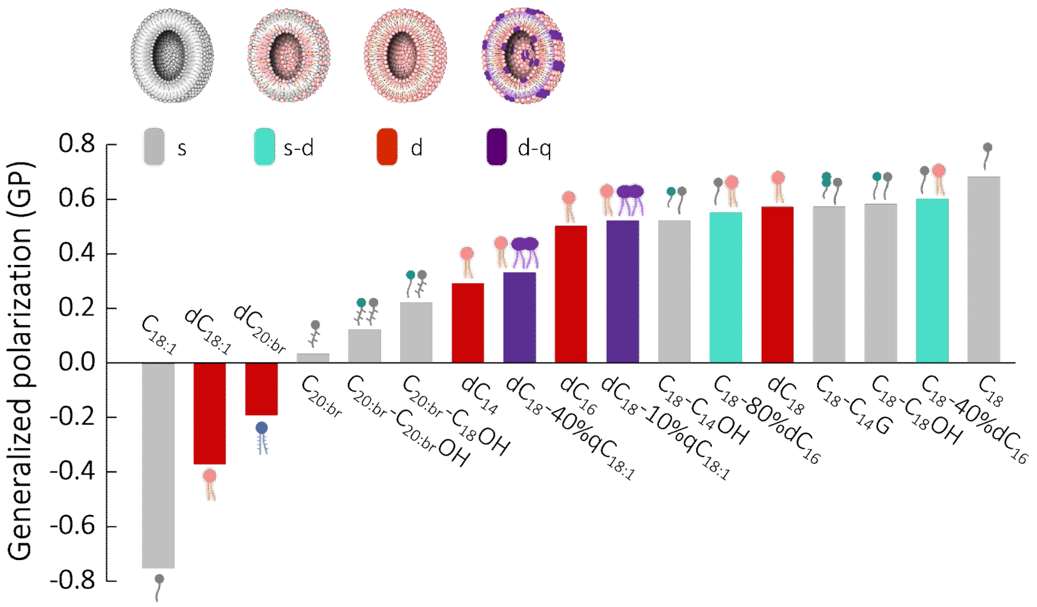


**Figure S11. The GP value of vesicles assembled with fatty acids/alcohols or phospholipids at room temperature (~20 °C).** The mole ratio of acid/alcohol is 2:1. s, single-chain (grey); s-d, single-chain and double-chain (cyan); d, double-chain (red); d-q, double-chain and quadruple-chain (purple).

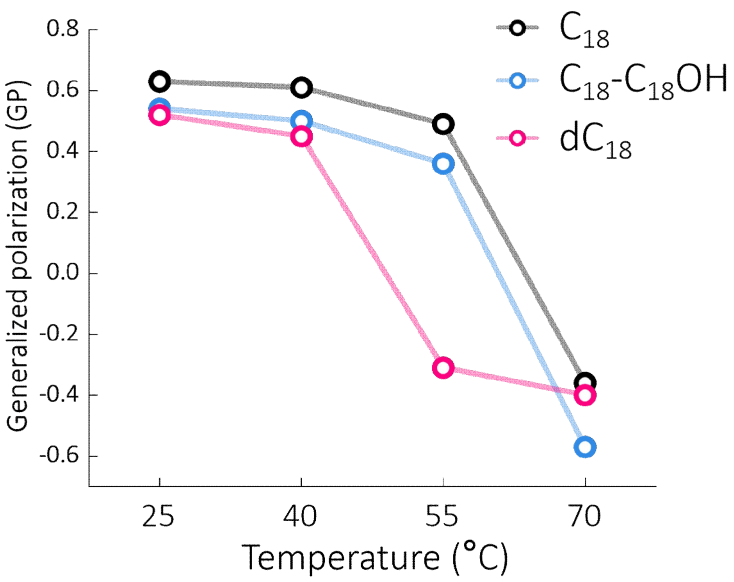


**Fig. S12 The influence of temperature on membrane fluidity.** The GP value of $C_{18}$, $C_{18}$-$C_{18}OH$ and $dC_{18}$ vesicles at 25 °C, 40 °C, 55 °C and 70 °C, respectively.

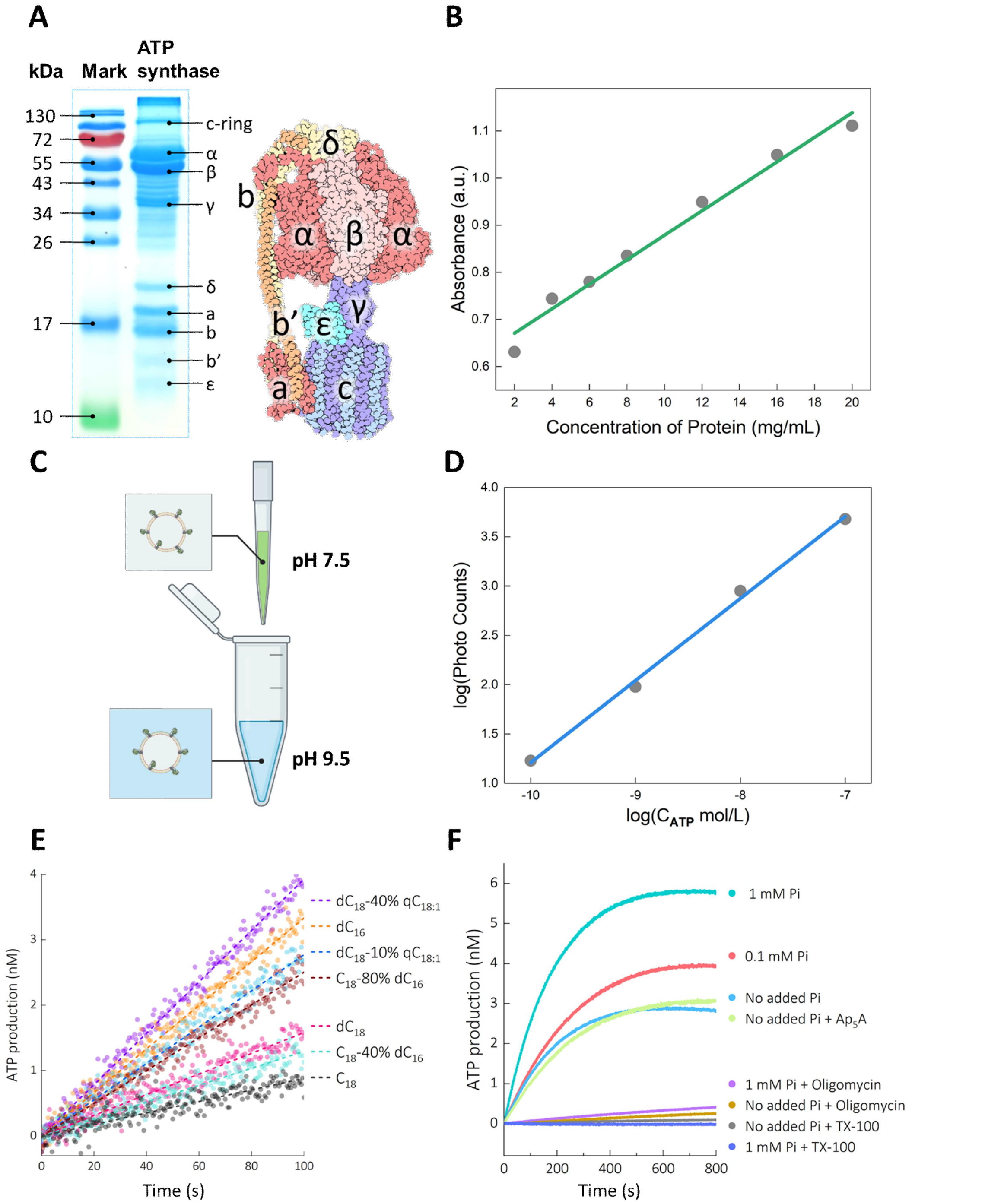


**Figure S13. Verification of structure and function of ATP synthase.** (A) The SDS-PAGE image of the extracted ATP synthase. (B) Standard curve used to determine concentration of ATPase synthase. (C) Schematic diagram of proton gradient formation by base bath. (D) Standard curve used to determine concentration of ATP. (E) ATP production of ATP synthase-reconstituted vesicles over time for calculating the relative apparent ATP synthesis rate based on the slope of the initial 100 seconds. (F) ATP production of ATP synthase-reconstituted $C_{18}$ vesicles over time. ATP synthesis is phosphate-dependent. When no additional phosphate (Pi) is added, ATP synthesis also occurred, because of the residual phosphate buffer in the ATP synthase extraction solution. Oligomycin is an ATP synthase inhibitor. TX-100 can break the vesicles. Ap5A is an adenylate kinase inhibitor.

## Supplemental References


1. Jordan, S. F., Rammu, H., Zheludev, I. N., Hartley, A. M., Maréchal, A. and Lane, N. (2019). Promotion of protocell self-assembly from mixed amphiphiles at the origin of life. Nat. Ecol. Evol. *3*, 1705-1714. https://doi.org/10.1038/s41559-019-1015-y
2. Budin, I., Debnath, A. and Szostak, J. W. (2012). Concentration-Driven Growth of Model Protocell Membranes. J. Am. Chem. Soc. *134*, 20812-20819. https://doi.org/10.1021/ja310382d
3. Li, Z., Yu, F., Xu, X., Wang, T., Fei, J., Hao, J. and Li, J. (2023). Photozyme-catalyzed ATP generation based on ATP synthase-reconstituted nanoarchitectonics. J. Am. Chem. Soc. *145*, 20907-20912. https://doi.org/10.1021/jacs.3c06090
4. Dezi, M., Di Cicco, A., Bassereau, P. and Lévy, D. (2013). Detergent-mediated incorporation of transmembrane proteins in giant unilamellar vesicles with controlled physiological contents. Proc. Natl. Acad. Sci. U. S. A. *110*, 7276-7281. https://doi.org/10.1073/pnas.1303857110
